# The struggle for life of the genome's selfish architects

**DOI:** 10.1186/1745-6150-6-19

**Published:** 2011-03-17

**Authors:** Aurélie Hua-Van, Arnaud Le Rouzic, Thibaud S Boutin, Jonathan Filée, Pierre Capy

**Affiliations:** 1 Laboratoire Evolution, Génomes, Spéciation. CNRS UPR9034/Université Paris-Sud, Gif-sur-Yvette, France

## Abstract

Transposable elements (TEs) were first discovered more than 50 years ago, but were totally ignored for a long time. Over the last few decades they have gradually attracted increasing interest from research scientists. Initially they were viewed as totally marginal and anecdotic, but TEs have been revealed as potentially harmful parasitic entities, ubiquitous in genomes, and finally as unavoidable actors in the diversity, structure, and evolution of the genome. Since Darwin's theory of evolution, and the progress of molecular biology, transposable elements may be the discovery that has most influenced our vision of (genome) evolution. In this review, we provide a synopsis of what is known about the complex interactions that exist between transposable elements and the host genome. Numerous examples of these interactions are provided, first from the standpoint of the genome, and then from that of the transposable elements. We also explore the evolutionary aspects of TEs in the light of post-Darwinian theories of evolution.

**Reviewers:**

This article was reviewed by Jerzy Jurka, Jürgen Brosius and I. King Jordan. For complete reports, see the **Reviewers' reports **section.

## Background

For a century and half, from the publication of "***On the Origin of Species by Means of Natural Selection, or the Preservation of Favoured Races in the Struggle for Life***" by Darwin [1] to the present day, thinking about evolution has not drastically changed, but it has itself "evolved" by taking on board new insights, and all the fresh data arising from the last 30 years of molecular biology [2]. This review focuses on the changes that have resulted from advances in our knowledge about the biology of transposable elements.

At the time Darwin published his ***Origin of Species***, chromosomes, DNA, genes, and heredity mechanisms were all totally unknown. There was considerable progress in all these domains during the 20^th ^century, which corresponds to the golden age of genetics. From Mendel to Watson and Crick, *via *Morgan and Weismann, Darwinian theory has evolved and successively integrated the laws of inheritance (neo-Darwinism), and then biometric, populational, ecological concepts (the modern synthesis, established between the 1930s and 1940s by Fisher, Wright, Haldane, Dobzhansky, Mayr, and Simpson among others), and finally the molecular dimension (Kimura's neutral evolution theory, Pauling and Zuckerkandl's molecular clock concept). However, the core of Darwin's theory has never really been successfully challenged.

The second part of the 20^th ^century was dominated by a fresh and powerful discipline, molecular biology, which claimed to explain the nature of life. This was dominated by a central dogma, which was rooted in the chromosomal theory of heredity, and the deciphering of the structure of DNA. The genome was envisaged as a stable structure consisting of DNA, from which switchable genes would transfer the genetic information necessary for the development or the survival of the organism to the relevant proteins. This idea held sway for many years, before it too was revealed to be an oversimplification of how genetic information is transferred [3].

At the onset of this exciting period, around 1944 at Cold Spring Harbor, the brilliant maize geneticist Barbara McClintock was using cytogenetic tools borrowed from Drosophila techniques, and was patiently investigating an odd phenomenon of chromosome breakage and fusion. Her painstaking observations and rigorous experiments led her to postulate the existence of a locus with a controlling element that was able to modify the expression of a gene at another locus. Subsequently she found that there were in fact several of these controlling loci, which were normally in a silent state, but which could occasionally be activated following genomic stress, such as a double-strand break. Moreover, the controlling locus was able to change its chromosomal location. She called this system Ac/Ds (for Activator/Dissociation), and designated the associated phenomenon of relocation "transposition". The first transposable element (TE) had been discovered, thus providing both the very first evidence of the impact of TEs on gene regulation, and the first indication of TE regulation by the genome.

### From incredulity to inescapability

The history of TEs is much shorter than the history of the theory of evolution: it is less than 70 years since Barbara McClintock first reported the existence of controlling elements. However, even though her discoveries were rigorously supported by experimental data it took much longer for McClintock's findings to gain acceptance than it had for Darwin's theory. Basics of Darwin's theory relied on common sense, and it was clearly its implications for evolution and the origin of the human species that aroused his virulent detractors. McClintock discovered transposable elements at a time dominated by the idea of genetic stability, which appeared to be essential for transmission to descendants, and for the conservation of species characteristics. The concept of genetic stability had emerged after Mendel's laws, and was later reinforced by discoveries such as the structure of DNA, and the regulation of bacterial genes. The established view of a static genome seemed to be unquestionable. Her work aroused a reception that may have been less hostile than that of Darwin's detractors, but her work was not understood, and gave rise to incredulity, rejection and sarcasm [4]. This lasted for several years and indeed decades, until the identification of similar elements in other genomes [5] began to win round the wider scientific community. Eventually the transposition of DNA fragments was demonstrated using the tools of molecular biology. In 1983 Barbara McClintock was finally awarded with the Nobel Prize for her discovery of transposable elements.

It is now no longer possible to ignore TEs. The impact of TE-derived sequences in regulating genes needs no further proof. Everyone accepts that genomes are quite flexible or plastic entities, that they are riddled with TEs, and that TEs affect both gene regulation and the composition and structure of the genome. The depiction of the genome as a linear succession of genes and the dogma of its stability have been replaced by a dominant view of a functional genome as a complex network of genetics, epigenetics, and cell interactions, in which TEs and other structural or functional elements are involved. 25 years after McClintock's Nobel Prize, have we fully embraced the full extent and diversity of the influence of TEs, notably in genome evolution?

### The astonishing properties of jumping genes

TEs possess two main characteristics that distinguish them from other genomic components. They are mobile, so able to change their genetic environment, and by doing this they also change the genetic environment of the locus into which they insert. Since they have the intrinsic ability to multiply during the transposition process, they are almost inevitably repeated, with a virtually unlimited copy number, restricted only by the carrying capacity of their environment *i.e. *the genome. Hence they are simultaneously part of the genome and independent entities living their own life within the genome, in a way that reminds Dawkins' selfish gene [6].

How can TEs be integrated as a major evolutionary factor in Darwinian theory? How do TEs influence genome evolution, and how does genome evolution influence TEs? Do they exploit the genome? Are they exploited by the genome? Are they parasites of the genome or part of it? What would evolution and life have been like without them? The answers are complex, because the interactions between TEs and their host genomes are complex. In this review we attempt to propose some clues to the answers to these questions. Some of the properties described below show how TEs fit in with the most recent developments in evolutionary theory.

#### 1 - TEs are a major factor in evolution because they are an important source of variability

Mutations caused by TEs are diverse, ranging from small-scale nucleotide changes (*i.e. *excision footprint) to large chromosome rearrangements, including epigenetic modifications. Although TEs are mobile, the nucleotide (or epigenetic) changes resulting from their transposition can persist, being transmitted through generations and through populations.

#### 2 - TE insertions are subject to natural selection

In a population, deleterious insertions (*i.e. *ones that reduce the host's fitness) will tend to be eliminated, whereas neutral and advantageous effects may be maintained, as are some other polymorphisms/mutations. This selection process occurs in the context of competition between individuals (genomes), but of course TE-associated genetic variation is also subject to other evolutionary forces, such as genetic drift or migration.

#### 3 - TEs multiply independently within the genome and consequently evolve more or less independently of the genome

In addition to this competition between individuals or genomes harboring TEs, competition also exists between TE copies that inhabit the same genome. TEs that are able to produce more copies have better chance of invading the genome and the population than those that rarely duplicate. Hence the dynamics of TEs includes two levels: an intra-genomic level, and an intra-population one. Furthermore, TEs frequently generate defective copies that behave like parasites towards the autonomous copies. Hence population genetics and ecological principles can be applied to a TE population within a genome. From this point of view, TE copies can be viewed as analagous to individuals, TE families to species, and genomes to ecological niches. Non-autonomous elements are assimilated to parasites. TEs can also occasionally transfer horizontally from one species to another. From the ecological point of view, horizontal transfer (HT) corresponds to the colonization of a new ecological niche. For the TEs, it constitutes another way to ensure survival.

#### 4 - TEs are involved in close interactions with the genome

Numerous long-standing and complex interactions have developed between TEs and host genomes, as a result of an arms race or of molecular domestication. Epigenetic phenomena may have evolved from ancient defense mechanisms set up by the genome to defend itself against foreign DNA (viruses or TEs). TEs may have evolved auto-regulation processes in order to limit the deleterious effects of uncontrolled transposition bursts. Genomes may have recurrently recruited TEs, parts of TEs, or TE-derived enzymatic or structural functions for its own purposes, drawing primary materials and ready-to-use tools from the numerous sequences comprising the TE.

The original vision of TEs as genome parasites was rather simplistic. In fact, TEs participate in the construction and evolution of the genome to an extent that would have seemed unbelievable until recently. TEs survive in the genome, feed on the genome, and feed the genome. TEs are probable an essential, long-standing part of the genome. This may contribute to their virtual ubiquity (with very few exceptions) among living beings.

## The TE landscape

### Structure and classification

Transposable elements exist in every known eukaryotic, bacterial or archaeal genome. They are defined as DNA sequences that are able to move from one chromosomal position to another within the same genome (i.e. within a single cell), which distinguishes them from phages and viruses, which move from cell to cell.

TEs usually encode the genes that promote their own transposition, but many non-autonomous elements use the transposition machinery of close relatives or unrelated elements instead. TEs are divided into two classes depending on their transposition mechanism, each class is further divided into subclasses, orders and superfamilies [7].

Class I elements transpose through an RNA intermediate, transcribed from DNA then reverse transcribed into double-stranded DNA (dsDNA) before or during their integration into a new position. They are replicative by nature. The key enzyme is a reverse transcriptase (RT), which is present in the telomerases of eukaryotes, but which is also an overall characteristic of mobile RNA entities (retroviruses, group II introns, and retrotransposons). RT is also present in bacteria, in elements such as retrons, group II introns and diversity-generating retroelements, although their mobility has been proven only for group II introns [8]. In Eukaryotes, four orders of autonomous retroelements are recognized [7], (i) Long Terminal Repeats (LTR) retroelements, similar in structure to retroviruses, (ii) Long INterspersed repeated Elements (LINEs), elements which have no LTRs but do have a polyA tail, (iii) DIRS (from DIRS-1, the first element identified in *Dictyostelium*) and (iv) PLEs (Penelope-like elements), these two last groups having somewhat unusual structures. In eukaryotes, several Class I non-autonomous elements have been identified. Short INterspersed repeated Elements (SINEs) are usually derived from tRNA and use LINEs to transpose. They may contain the 3' part of LINEs, probably fused to the tRNA at the time of retrotransposition [9]. All other non-autonomous retroelements possess typical structural features or are deletion derivatives of one of the four orders of autonomous retroelements (LTR, LINE, DIRS, PLE).

The diversity of retroelements reflects their complex origin. Indeed, phylogenies based on RT suggest that LINEs are related to group II introns, and that most retroviruses belong to one superfamily within the LTR order, despite several independent examples of infectious retroviruses originating from LTR-retroelements [10]. However, phylogenies based on other protein domains (endonuclease or RNAseH) display different topologies, suggesting that the various retroelements originated from independent fusions of different modules [10,11].

Class II elements transpose directly with no RNA copy intermediate. They can excise from the donor site (they are known as cut-and-paste transposons, and the transposition is described as conservative) although this is not always the case, since several Class II elements are replicative (i.e. their transposition is coupled with replication). Hence, Class II has been divided into two subclasses depending on the number of DNA strand cuts at the donor site, which reflects these different transposition mechanisms. In the subclass I, the two strands are cut at both sites, and the element is fully excised [7]. This subclass comprises mainly those elements that are characterized by having two terminal inverted repeats (TIR) and at least one gene encoding the transposase (TIR elements Order). They are especially abundant in prokaryotes, where they are known as insertion sequences (IS), and are also widespread and diversified in eukaryotes. On the basis of transposase similarities, TIR elements can be divided into 12 to 17 superfamilies in eukaryotes [7,12,13], and more than 20 in prokaryotes [14,15]. However, a number of prokaryotic and eukaryotic superfamilies are related and thus form trans-domain superfamilies, which suggests that these superfamilies are either old enough to have preceded the split into the three domains of life, or that horizontal transfers occurred in the distant past [16]. Subclass I also includes elements that do not possess a transposase, but instead have a recombinase that is able to recombine two DNAs without generating free ends. Recombinase-containing Class II elements are frequent in prokaryotes [14], and have also been found in eukaryotes, although so far only in some opisthokonts (crypton elements) [[17], and see RepBase http://www.girinst.org]. When only one strand is cut on each side, the transposition is said to be replicative. In eukaryotes, two recently discovered types of Class II elements (Polintons/Mavericks and Helitrons) are thought to transpose in such a way. Polintons are very large elements, bordered by TIRs and containing several genes, including an integrase (related to retroviral integrases and Class II transposases) and a polymerase [18]. Helitrons are moderately large, possess hairpin structures at the ends, and contain a helicase [19]. These characteristics are reminiscent of a rolling-circle mechanism, such as that involved in IS91. In bacteria, another recently identified family (IS608) is characterized by having a transposase related to the RCR protein of IS91, which recognizes specific secondary structures, such as hairpins, at the tips of the elements. However, the transposition mechanism seems to be different [20]. Finally, prokaryotes also carry more complex TEs-based structures that trap a large range of mobile genes, such as in composite transposons (Tn) or in Integrative and Conjugative Elements (ICEs) [21], illustrating that evolution can also occur by modularity [22].

Prokaryotic and eukaryotic TIR elements frequently generate considerably reduced non-autonomous elements known as Miniature Inverted repeat Transposable Elements (MITEs) that use a transposase encoded *in trans *to transpose. MITEs are either deletion derivatives of full-length elements, or only share TIRs with their autonomous partner. Helitrons are also often found as non-autonomous copies (derived from an internal deletion).

### Abundance and distribution

**Figure 1:**
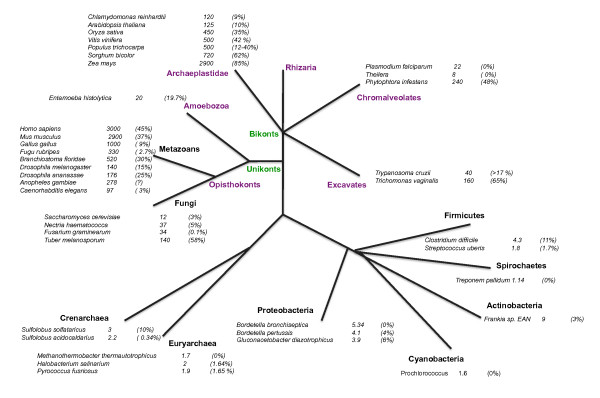
**TE contents in various different sequenced species: genome size are in Mb**. TE percentages are shown within parentheses. References can be found in Additional File 1.

The abundance of TEs in each eukaryotic and prokaryotic lineage is highly variable (Figure 1 and Additional file 1). TEs are more ubiquitous in eukaryotes (most genomes contain TEs) than in prokaryotes, in which more that 20% of the genomes so far sequenced lack both remnants and complete TEs [23]. Furthermore, TEs are far more abundant in eukaryotic genomes (comprising up to 80% of the genome) than in prokaryotes (up to 10% of the genome, averaging only 1-5%). However, in both prokaryotes and eukaryotes, there seems to be a positive correlation between genome size and TE abundance [23,24]. Retroelements (that have intrinsic replicative properties and may be large in size) are often the main provider of TE DNA in eukaryotes, such as several mammals, yeasts, Drosophila, and plants with large genomes [25-27]. In some cases, however, (e.g. *Trichomonas vaginalis, Caenorhabditis elegans*), Class II elements dominate, at least in terms of copy number [16]. In contrast, small eukaryotic genomes (parasitic apicomplexa for example) are usually devoid of TEs, perhaps because of a general tendency towards genome size reduction.

In many genomes, a few elements dominate, but this does not preclude an extraordinary diversity, which is usually in the range of hundreds of element families. For example, LINEs of the *L1 *type and SINEs *Alu *are predominant in the human genome, and diversified as a few subfamilies of different ages. In contrast, LTR retroelements (endogenous retroviruses) are found in relatively low copy numbers, and belong to a few dozen different families [26]. So TE diversity and abundance is highly variable from one species to another, and reflects their specific genome-TE history. In addition, TE distribution within a genome is usually neither random nor uniform. First some (rare) elements are site-specific, such as LINE R2 elements, which exclusively insert into a single site in the rDNA, or some IS elements in prokaryotes. Secondly, TEs are frequently found in chromosomal regions where their potentially deleterious impact is reduced. Thus, TEs are common in the heterochromatin and in pericentromeric or telomeric regions, in low gene density regions, or within other elements. However, they can also be found in open chromatin regions, near tRNA genes, and near promoters and genes. Hence, in some plants DNA transposons are preferentially found around genes.

This non-random distribution, including an apparent preference either to insert near to genes or to avoid them, may result either from a true insertion preference or just from selection. The study of recent insertions obtained in the lab or in the wild may help distinguish between these two hypotheses [28].

Our view of TE landscape is biased, because genome sequencing efforts have mainly focused on bacterial genomes or on the "higher eukaryotes", and so are not representative of the full diversity of life. Moreover, TE contents can differ greatly between closely related species. For example, the Archaea *Sulfolobus solfataricus *carries at least 130 full IS copies and at least 200 partial IS copies, whereas the related species *Sulfolobus acidocaldarius *carries only a few partial IS copies [14,15]. Furthermore, differences are often observed between strains of the same species in terms of copy number [28,29]. As the number of genome research projects increases and technology progresses, we can hope that the representations of the tree of life and of intra-species diversity will improve. This may well reveal that TE history has as many versions as there are populations bearing them.

### Consequences of TEs at the DNA level

The presence of TEs (as dispersed, mobile, and repeated elements) has two major mutational consequences at the DNA level: insertion within a locus, and ectopic recombination leading to different types of rearrangements. First, TEs can insert near or within genes, and by doing so alter or destroy the activity of the gene in a variety of ways, ranging from total inactivation to spatio-temporal changes in expression, alternative splicing, or changes in expression level or protein activity. Modifications of gene expression can be a direct consequence of adding extra nucleotides to the original sequence or an indirect consequence of the epigenetic marks on the element. Furthermore, in addition to promoter and terminator sequences, TEs sometimes carry silencers or insulators that are able to modify expression over distances of several kb, or binding sites for different proteins (*i.e. *heterochromatin protein) [30-32]. Second, the possibility of recombination between two copies at different loci can also have a more or less dramatic effect, ranging from small-scale inversions to major chromosomal rearrangements, including deletions, translocation or duplications [33,34].

Although TEs are defined as intracellular parasites/entities, they are prone to being transferred from cell to cell, notably in prokaryotes through conjugation of the element or of the plasmids carrying it. A major consequence is lateral gene transfer (LGT), also known as horizontal transfer (HT), which is quite common in bacteria. In eukaryotes, numerous cases of TE HT have been reported, although the vector involved remains elusive. Interestingly, several TEs have been found in eukaryotic viruses, such as TED, *piggyBac*, or *Tc1*-like in baculoviruses [35], DIRS elements in a Polydnavirus genome [36] or TEs related to the IS605/IS607 family in Phycodnavirus and Mimivirus genomes [37]. Thus, these viruses could be used by TEs as a potential source of horizontal dissemination in eukaryotes.

### TEs and the genome: an evolutionary point of view

The way we imagine genome evolution today has not departed from the Darwinian theory. Any new gene, or new function, which confers advantages in the host in a given environment, will be selected as long as the host is in this environment. This is true for the selfish genes of Dawkins: any gene that is able to propagate successfully (by vertical transmission) in a given environment (the genome, including other genes) will successfully disseminate in this environment [6]. This also holds on for TEs, which can be viewed as selfish DNA: the ultimate parasite [38], which is able to propagate itself through vertical transmission, through intra-genomic transposition, and through horizontal transfer.

TEs are able to replicate more rapidly than the genome, and so constitute a kind of genomic cancer. They are basically parasitic, i.e. selfish, and deleterious entities, conferring no benefits on the genomes they inhabit. For these reasons, they were long considered to be "junk DNA", part of the genome that by definition it would be better to get rid of, because it has no role, no function, and is just a kind of genetic burden for the host genome. This simplistic view must now be tempered. First of all, TEs and the rest of the genome have lived side by side for a very long time and such prolonged co-habitation almost inevitably leads to various kinds of interaction. Second, having no known role does not necessarily imply having no impact: day after day, portions of the genome that were previously thought to be useless have been shown to have important regulatory or structural roles. The same could be true for TEs. Hence, when considering genome evolution, TEs are far from being just parasitic sequences [39]. Starting from the simple assumption that DNA is separated into two compartments, the genome, and the TEs, we review below the relationships between them in all their diversity. We will focus first on how the genome deals with the sea of TEs that surrounds it, and then on how TEs deal with the host genome in which they are embedded.

## Evolution of the genome in a sea of TEs

Although Darwin had no idea about what constituted the support of heredity, he fully recognized the importance of variability as the raw material of natural selection. It was a long time before connections could be established between continuous variation in a population, the discrete characters Mendel used to demonstrate the laws of heredity, and mutations (as defined by de Vries) as progenitors of new varieties. After these solid bases had been established, even McClintock could probably not imagine that the complex phenomena she was studying, which clearly defied the Mendelian laws, would later turn out to be such a key element in genome evolution.

### For the genome, TEs are a major source of genetic variation

From mutations to polymorphism, genetic variants reflect the diversity within a population, and DNA alterations or changes constitute the basis of evolution. At the DNA level, two molecular mechanisms are responsible for generating diversity: mutation and recombination. Classically, mutations (changes in nucleotide sequences) arise either through uncorrected replication errors or after DNA lesions; whereas recombination is a normal process during the meiotic phase. However, both processes can also result from the activity or mere presence of a transposable element. Transposition does not result from fortuitous errors during replication or lesion repair, but can be considered to be an active mutagenic process, resulting in mutations that are different from SNPs (Single Nucleotide Polymorphisms). In contrast, TE-induced ectopic recombination can be viewed as an erroneous (albeit easy-to-produce) process in contrast to normal meiotic crossing-over.

Such DNA alterations may affect the function (of genes) and the structure (of genomes), the worst outcome being the immediate death of the cell, or its inability to complete meiosis. However, mildly detrimental or neutral effects are also to be expected, and insertions that produce such effects may survive and contribute to the genetic variation of the host genome. There are many diverse ways in which TEs can alter the genome, ranging from small sequence modifications to gross rearrangements. Finally, the frequency of such events is not negligible, which means that TEs are major actors in diversity [40,41].

#### 1 - Genomes use TE sequences and TE-induced sequence changes to increase their functional variability

From a functional perspective, genetic variations imply changes in gene regulation (through sequence changes in a regulatory region, or epigenetic changes), changes in coding sequence, or a change in splicing. Any such genetic variations can be the result of TE activity, involving insertion, excision, or ectopic recombination [42,43]. Genetic variations in the genes can result in phenotypic changes, which are easy to detect and investigate. Hence genetics has tended to focus on transmissible, visible, and discrete variations between lineages. One of the characters used by Mendel to establish the transmission laws was the stable phenotype of wrinkled peas (versus smooth peas). For Mendel, the stability of the phenotype was a prerequisite he had carefully checked before selecting his experimental characters. Amazingly, this stable character ultimately turned out to be the result of the insertion of a (non-autonomous) TE within the *s *gene [44]. Even before their discovery, TEs were under the spotlight! Class I elements, as well as on-autonomous elements without their autonomous partners, will not usually excise from their position, which means that the altered phenotype is stable (however, see below). In contrast, autonomous class II elements are recognized as triggering phenotype instability. Moreover, phenotype reversibility has proved to be an effective criterion for identifying active DNA transposons [45]. Unstable mutations (resulting in variegation or mosaicism) were already known when McClintock started working on the chromosome-breaking cycle, and some of these cycles were associated with this lack of stability. What she found was that this instability was controlled, since the mutation rate was constant within a given plant [46].

In Eukaryotes, visible polymorphism often results from the action of TEs. Numerous examples involve color polymorphism, and TEs. In morning glory (*Ipomoea spp.*), the petal color polymorphism is caused by various transposable elements that have been inserted into genes involved in pigment biosynthesis [47,48]. Alternatively, somatic TE excision (usually imprecise) can also result in phenotypic changes, responsible for variegation, spots or sectors. Hence in snapdragon (*Antirrhinum maju*s) the imprecise excision of *Tam3 *from the *pallida *gene results in diverse spatial color patterns [49], as in Medaka fish, in which the excision of *Tol2 *inserted in the promoter of a pigment gene generates numerous phenotypes distinct from original mutant or wild-type [50]. Phenotypic variations due to TEs can also affect other traits, as exemplified by the recent identification of a TE-induced duplication, which is responsible for the elongated shape of a tomato [51], or the impact of TE insertions on Drosophila bristle numbers [52]. Finally, a epigenetic component may be involved in many TE-mediated phenotypic variations [41].

In prokaryotes, there are fewer examples of changes in gene regulation associated with TEs, but some IS elements have been shown to be involved in the versatility of some systems. A striking example is the *Staphylococcus aureus IS256*-mediated switch between the ability and inability to form a biofilm [53,54]. This IS is involved in about 30% of the cases, but nevertheless, insertions appear to occur as random, uncontrolled events. In the much-studied *Mycobacterium tuberculosis*, the highly mobile element *IS6110 *seems to be a major factor in strain diversity, phenotypic alterations, and thus in evolution [55]

#### 2 - TEs as genome architects

In addition to their influence on the functional compartment of the genome, TEs are also involved at the structural level, and are an important factor in the genomic peculiarities of species. However, modifying the genome structure will inevitably lead to functional changes. From this point of view, TEs are a key that links the structure and function of the genome.

Beside polyploidization, TEs are the major factor of genome expansion. Intensive TE transposition provides an explanation for the "C-value paradox", i.e. the fact that in eukaryotes, genome size is not correlated to the complexity of the organism, or to the gene number [24]. In plants, bursts of transposition of retroelements have been shown to be responsible for the genome size expansion [56,57], and every large genome is expected to harbor a huge number of TE sequences. On the other hand, by promoting gene inactivation and recombination-mediated chromosomal deletion, TEs can also be involved in genome simplification. In prokaryotes, TEs seem to be associated with the drastic reduction in genome size observed in some *Bordetella *and *Yersinia *species [58,59].

In eukaryotes, transposable elements are not distributed randomly along chromosomes. They are particularly abundant in constitutive heterochromatin, notably in centromeres and telomeres. Centromeric TEs either constitute the core sequences of centromeres or are merely centromere-specific [60-62], and may be found as intact or fragment tandem repeats [63]. This suggests a direct role in centromere function, and in the generation of satellite sequences. They are also frequently found in pericentromeric regions [64,65], and in heterochromatin [66], and so they could also be involved in heterochromatinization [67], which links them to epigenetic regulation [68]. In numerous species, a similar pattern is observed near telomeres. TEs enrichment in telomeric and subtelomeric regions has been found in diverse species of fungi, vertebrates, insects, protozoa, or plants [69-73]. Telomeric TE accumulation may result from relaxed conditions in those regions, as TEs have no known function, with the exception of the LINE elements in Drosophila, which replace telomerase (see below)[74].

The role of TEs in genome compartmentalization was suggested after the discovery of TEs in scaffold/matrix attachment regions (S/MARs) that determine chromatin loops [75,76]. In plants, this mainly involves MITEs, which are AT-rich like S/MARs [77], but Jordan et al. [76] also found that LINEs were overrepresented in human S/MARs. In Drosophila, the insulator (aka *su*(*Hw*)) of the *gypsy *retroelement (*mdg4*), has been extensively studied for its role as an enhancer blocker, and may function as an S/MAR [see [76]]. This constitutes the best-documented example of a TE that lies at the junction between structural and functional roles.

Although TEs are usually silent, bursts of activity and high TE copy number can lead to rapid genome diversification between close species, as a result of lineage specific amplification or recombination [78]. Some authors have even suggested that TE-mediated gross rearrangements may be involved in speciation. The first person to do so was Barbara McClintock herself [79-81]. However, this still remains speculative, and we have no evidence of a direct cause-effect relationship between TE transposition or recombination and speciation. In Drosophila, the phenomenon of hybrid dysgenesis is directly related to the activity of particular TEs (*P*, *hobo *or *I*), and results in cross incompatibilities between some strains, a potential first step towards reproductive isolation [82].

When an increasing proportion of the DNA consists of TEs, new insertions, even if they occur randomly, will be more and more likely to occur within another transposable element, thus creating and expanding TE clusters. Moreover, as insertions within other TEs are usually selectively neutral, they have less impact on host fitness, and so no selection is exerted against them, leaving them free to accumulate in clusters. Although TEs are thought to accumulate in low recombination regions [83], regions rich in TEs are usually more unstable, and more prone to illegitimate recombination [84].

Genomic variability at the location of mobile DNA is also observed in prokaryotes, in which composite transposons (Tns) and Integrative and Conjugative Elements (ICEs) occur, and appear to be prone to exchange, gain or lose gene modules, probably through nested insertions and rearrangements [21]. This is illustrated by the recent finding that in *Helicobacter pylori*, plasticity zones, containing strain-specific genes, actually consist of a mosaic of several ICE, Tn and IS elements [85].

Prokaryotic elements that are able to gain or lose gene modules are a good example of how mobile elements contribute to the genome content, but ICEs are usually site-specific, and so do not amplify within a single genome, but are transferred from cell to cell by conjugation [86]. However, in some cases, ICEs have undergone massive expansion, such as the 185 ICE elements in *Orientia tsutsugamushi *that occupy 35% of the genome [87].

In eukaryotes, host gene sequences have been found in some TEs, notably Pack-MULEs and Helitrons in plants [88]. By amplifying, these elements spread these genes or gene fragments throughout the genome (sometimes as chimeric variants), which results in opportunities for gene duplication and exon shuffling. Both can be promoted through TE-mediated (illegitimate) recombination [88], or by class II elements engaging in a complex transposition process, known as aberrant transposition [89]. Aberrant transposition, which uses several transposons and results in various orientations, was the kind of transposition event observed by McClintock in the maize chromosome break-fusion-bridge fusion. Although illegitimate recombination and aberrant transposition are "abnormal" processes, their consequences may have an important impact, since gene duplication and exon shuffling are major processes in gene evolution [90].

#### 3 - From mutations and (epi)-genetic variation to genetic novelty and adaptation

In the past, it has often been suggested that TEs have detrimental effects, as TEs were often viewed as deleterious parasitic entities [6,38,91]. In fly, it was estimated that 80% of spontaneous mutations resulted from transposable elements [92]. In other species, the estimate is considerably lower: 15% in mouse [78], whereas in human only 0.5% of genetic diseases are caused by TEs [93], and most human TEs are currently inactive.

However, population and polymorphism studies suggest that TEs activity often has a neutral or near-neutral effect. TE insertion polymorphism is common enough to provide an efficient tool for strain typing, population studies and phylogeny [94,95], and far more representative of the genetic diversity than phenotypic polymorphism. In human, a recent study intended to quantify such polymorphism detected at least 600 *Alu *polymorphisms, and suggested that human populations may bear up to 2000 TE polymorphisms [96] - far fewer than SNPs, but still a significant number. In cultivated rice, more than 50% of large insertion/deletion events involve TEs, and TEs account for 14% of the genetic difference between strains [97]. If an insertion is neutral, its persistence in the population relies on genetic drift and demographic parameters or occasionally on hitchhiking from a close locus under positive selection, and is thus perfectly compatible with Kimura's neutral evolution theory.

TE insertions can sometimes have beneficial effects. Several putative cases of adaptive insertion have been detected by population and site occupancy frequency studies [98]. However, the reason why insertions are beneficial remain unknown [99,100]. In some other cases, the effect of the insertion is more obvious, such as the increased resistance to insecticide of Drosophila strains with a *Doc *element within a P450 gene [101]. Finally, TEs may sometimes be involved in important processes, such as those suspected for L1 elements in X inactivation [102]. Such cases may ultimately lead to molecular domestication processes, which will be described in more detail in the third part of this review.

Genetic variation is the playground in which natural selection plays. Hence, TEs, by increasing their variability, increase the adaptability and evolvability of genomes and species. Divergence studies suggest that TEs proceed by successive amplification bursts [[103,104] for examples]. By analogy with radiation bursts observed in paleontology, they have been linked to evolution through the theory of punctuated equilibrium developed by Eldredge and Gould [105]. Hence, in certain well-studied vertebrate groups, TE activity has been detected at different times, which correspond roughly to periods of species diversification (notably in primates and bats). The direct role of TE activity in species radiation, defended by Oliver & Greene, and Zeh et al. [106,107], takes into account the fact that TEs are controlled in a reversible way by epigenetics (see below), are induced by stress, and that TE activity increases the genomic variation, thus resulting in better adaptability when conditions change.

### For the genome, TEs are disturbing invaders but can also be useful helpers

Epigenetic control is widely used by multicellular organisms, such as higher metazoans or plants, to implement cell lineage-specific gene regulation, and more generally for any developmental process, including X inactivation, parental imprinting, cell cycle, germ line development, and early embryogenesis [108-112]. Epigenetic mechanisms are also used to silence transposable elements, thus avoiding the detrimental effects of transposition. The present-day view is that epigenetics was first used to defend the genome against invading DNA (including TEs) before being exploited at a larger scale for gene regulation. The relationship between TEs, epigenetics, and gene regulation is in fact far more complex than this. TEs may have acted primarily as evolution drivers that led the genome to evolve defense mechanisms, and then gene expression control systems. Although present-day epigenetic gene regulation appears at first sight to be free of TE intervention, silenced TEs can nevertheless directly interfere with the expression of adjacent genes [68]. Furthermore, it has recently been proposed that TEs could ultimately have been exapted for regulation purposes [113]. Finally, occasional disruption of epigenetic control may offer an opportunity to enhance the evolvability.

#### 1 - The various epigenetic processes

Basically, epigenetic marks refer to DNA methylation of cytosine, to histone modifications at their N-terminal region *via *methylation, acetylation or phosphorylation, or to RNA interference through small RNAs (RNAi). Those modifications silence TEs either transcriptionally (TGS) by DNA methylation or as a result of changes in chromatin structure, or post-trancriptionally (PTGS) through small interfering RNAs that are able to destroy mRNA. In fact all three epigenetic mechanisms seem to rely (at least in part) on the same basic RNAi process [68].

DNA methylation is widely used to regulate expression. However, its importance varies considerably depending on the species, with methylation covering a large fraction of the large genomes of vertebrates and plants, whereas it is restricted in other metazoans and fungi [114,115]. Methylation in plants and fungi mainly targets TEs (or more generally repeated sequences), pinpointing this epigenetic mechanism as a defense against transposons. Independently of TEs, genes may also be methylated, even in the core gene region, thus permitting tissue-specific regulation [116]. However, while TEs are methylated through *de novo *methylation, gene methylation usually corresponds to maintenance methylation, and can be lost from time to time [117]. In vertebrates, TEs are globally methylated, as is the rest of the genome, which makes it less clear whether TEs are in fact specifically targeted by methylation [114]. The specificity of TE-targeted DNA methylation depends on the presence of short RNAs.

The chromatin state plays an important role in gene activity. In animals, this is particularly prevalent in all developmentally-controlled regulations [118]. The chromatin state is mainly regulated through histone modifications, such as the methylation or acetylation of histone's tail. These modifications can have repressive or activating effects on gene expression. Histone modifications are mediated by several protein complexes, which target specific sequences through interactions with gene promoters and transcription factors [119,120]. However, compelling evidence shows that RNAi is also an effector of chromatin modification, and is involved notably in transcriptional silencing and in heterochromatin formation at transposon sites [121-123]. Furthermore, DNA methylation and histone modifications are tightly interconnected [124-126].

Co-suppression in plants and quelling in fungi, were independently uncovered during the 1990s, after observing null phenotypes when transgene overexpression had been expected [127]. In *Caenorhabditis elegans*, a germline-specific process resulting in TE silencing was discovered in the 1990s and was termed RNA interference (RNAi) [128]. All these phenomena correspond to a gene-silencing mechanism (Post-Transcriptional Gene Silencing, or PTGS) that relies on short, non-coding RNAs (ncRNAs), and are generically known as RNA interference. RNAi exists in nearly all eukaryotes (with the notable exception of baker's yeast *Saccharomyces. cerevisiae*), albeit with variations and specificities. Moreover, several systems can be found within a single genome, which reflects evolution towards more specialized pathways. Different systems use different combinations of proteins from the same multigenic families (including the famous Argonaute family).

RNAi is the central key of epigenetic control, as it confers the necessary sequence specificity, and exists in different versions within and between species. For example, in Drosophila, three distinct pathways coexist, and generate siRNAs, microRNAs (miRNAs) and piwi-interaction RNAs (piRNAs - also known as rasiRNAs or repeat-associated siRNAs), respectively. Plants lack the *piwi *pathway, but their epigenetic systems are nevertheless quite diverse. These pathways differ by the origin of the processed RNA, its final structure, and the proteins involved in the whole process. However, the short RNAs produced always guide an Argonaute complex to the complementary nucleic acid for cleavage, translation inhibition, or chromatin modification [129].

The siRNA pathway is mainly a defense system against viruses, as siRNAs are generated from exogenous dsRNA. This leads to the destruction of transcripts. piRNAs are derived from long transcripts of transposon-rich genomic loci. piRNAs are targeted to repeated sequences, including TEs, and the silencing process involves an amplification cycle (ping-pong), and acts through RNA destruction, epigenetic modification of the homologous DNA locus, and the formation of heterochromatin. The piwi-pathway is germline specific, and in several species seems to correspond to a genomic defense against transmissible (germline) TE invasions. Indeed, in Drosophila and Zebrafish *(Danio rerio*), most piRNAs have homologies with TEs. However in mammals, most piRNAs do not correspond to TE sequences [130]. Finally, miRNAs arise from endogenous RNA (genomic locus), and are primarily used to regulate gene expression although some miRNAs are also derived from TE sequences. Hence, the miRNA system appears to have evolved from the defense systems to take on a gene regulation role.

#### 2 - Epigenetics as the genome's defense mechanisms against genomic parasites

The presence of invading selfish genes does not lead to a peaceful situation. Genomes have to fight against invasions that could lead to rapid reductions in fitness. This can be done in different ways. First, the genome may get rid of invading TEs by recombination, but this passive process may turn out to be less efficient than transposition. Second, the genome may inactivate TEs through targeted mutations. Such a process has been described in *Neurospora crassa *and other fungi, and is known as RIP (Repeat-Induced-Point mutations). It is quite efficient, at least in *N. crassa*, in the genome of which no intact TEs or TE activity can be detected [131]. The drawbacks are that the genome loses the benefits of TEs as a source of variations, and the benefits of having multigenic families - although in some conditions RIP may accelerate allele evolution [132]. Third, the genome may silence TEs epigenetically without destroying them. This is an efficient process, and one that has the advantage of being both transmissible and reversible. The potential source of variability (TEs) is still present in an inactivated state, but may occasionally be reactivated. Bursts of amplifications seem to have repeatedly occurred in the history of some genomes, and reflect periods when TEs escaped from epigenetic control [81]. In this system, TEs serve as a potential reservoir for future variability. TE silencing occurs by means of epigenetics, which is universally used in eukaryotic genomes, and has been particularly thoroughly investigated in plants [133]. Hence, present-day epigenetic systems (at least some of them, such as miRNA) are assumed to have evolved from systems originally set up to combat and limit the expansion of foreign sequences. The frontier between systems involved in gene regulation and those involved in TE silencing is not clear. Indeed, a number of important cellular processes are regulated through systems that borrow proteins used in the RNAi defense against transposons, such as PIWI in the germline [130].

#### 3 - The contribution of TEs to genome control

The contribution of transposable elements to the epigenetic phenomenon has recently been unraveled, but had long been suspected since McClintock proposed the existence of controlling elements as a response to environmental (or genomic) stresses [79]. From anecdotal "disturbers", TEs have now moved centre-stage and revealed to contribute to genome regulation and genome robustness and/or evolvability [68,134].

Transposable elements seem to occur in regions in which a concentration of epigenetic landmarks can be observed, and are often the target of the epigenetic control [68]. This may have two impacts: first, TE silencing; second, modification of the expression profile of nearby genes. While TE silencing will avoid amplification bursts, thus promoting a degree of stability, the silencing of genes in their vicinity may have an impact on the host [135]. More intriguingly, there are numerous examples suggesting the implication of TEs in the normal epigenetic regulation of genes, including genes involved in various developmental processes [68,109,136]. The assumption that TEs also contribute to regulation *via *intrinsic regulatory properties through nucleosome binding and phasing, epigenetic enhancers and boundary elements [137] constitutes a further step. Finally, TEs may have been exapted for these regulatory properties. Few studies have focused on histone modifications at TE sites, and the relationship between them remains poorly understood. In mammals, different TE classes seem to be targets for different histone modifications. However, contradictory findings make it difficult to work out whether histone modifications at TE sites result from a genomic defense or from exaptation for the regulation of adjacent genes [113].

It has long been known that a number of elements seems to reactivate following various stresses [138-140], and stress responses of retroelements are well documented in plants [141]. In Capy *et al. *[142], it was assumed that environmental changes can directly affect TE activity through the fixation of transcription activators on the regulatory region of the elements. It is now clear that TE reactivation by stress or environmental changes usually involves epigenetic changes [107]. Since the epigenetic state of TEs also influences the expression of adjacent genes, the reaction of the genome to stress directly involves TE sequences. In this case, TE-driven epigenetic control does not require the element to be active, since non-autonomous, deleted, truncated, and even dead elements can be subject to epigenetic marks. Hence, the most important point for the impact of the "epi-transposon" is the location of the insertions.

The combination of these different points suggests that we need to revisit the relationship between stress and TEs. TE reactivation (and the generation of variability) is not the only consequence to be expected after stress. Changes in the gene expression profile caused by epigenetic changes in neighboring TEs may also be of crucial importance. Both active and inactive TEs can have this effect, and so all types of copies must be considered. Given the existence of transgenerational inheritance, it is urgent to carry out theoretical and experimental investigations in order to define the impact of epigenetic phenomena induced by transposable elements at the population level. Very little has so far been published in this field, but in terms of evolution this is probably a key point [143,144].

#### 4 - Ancient origin of the components of RNAi

From an evolutionary point of view, the siRNA pathway, which is directed against both viral and exogenous RNAs, is the perfect example of a host-parasite arms race. Indeed, the RNAi defense is sometimes by-passed by various viral RNAi suppressors (VRS). Moreover, viruses have evolved ways of interfering with the endogenous miRNA pathways, allowing them to control host gene expression [145]. The host defense system has become very efficient by acting at both transcriptional and post-transcriptional levels, in both exogenous and endogenous sequences, and through ping-pong mechanisms or systemy (in plants and nematodes) [146]. The arms race is also illustrated by the rapid evolution of proteins involved in defenses against viruses and TEs, which contrasts with the slow evolution of the endogenous miRNA pathway proteins [145].

The RNAi system seem to have arisen in the common ancestor of all eukaryotes, since homologues of all three proteins involved in RNAi (the ARG family, DICER and RdRP) can be found in all the supergroups in which complete sequences exist (5 out of 6) [147,148]. Such a hypothesis looks likely when we recall that viruses and TEs are probably as old as life itself. More interestingly, homologous proteins also exist beyond the domain of the eukaryotes, although a prokaryotic origin of the RNAi system itself seems unlikely. Indeed, the RdRP and Dicer RNAse III domains may have evolved from phages, while the Dicer helicase domain and ARG/PIWI appear to originate from the Archaea. The roles of these prokaryotic proteins are not clear, but may not have been to defend the organism against foreign DNA [148], although alternative explanations have been proposed recently for the prokaryotic Argonaute proteins [149]. In any case, prokaryotes have other defense systems, with a different origin, but with somewhat surprising similarities. Apart from the widespread Restriction/Modification (R/M) system that specifically methylates endogenous DNA to protect it from degradation - note that R/M systems are also viewed as selfish modules [150,151] - prokaryotes also have Clustered Regularly Interspaced Short Palindromic Repeats (CRISPR) elements. These elements function *via *small RNA molecules to confer acquired immunity, in a way that may recall piRNA clusters. Parts of sequences from foreign mobile genetic elements, such as phages, or plasmids, are integrated into CRISPR regions between palindromic repeats. They are further transcribed and processed as small RNAs. These small RNAs serve as guides for a protein complex that targets the invading DNA [152]. Despite their striking functional analogy, eukaryotic RNAi systems and the CRISPR system are not phylogenetically related [153].

#### 5 - Impact on evolution

During the last decade, there has been an expansion in investigations of the molecular mechanisms underlying epigenetic phenomena. It has become clear that epigenetic components exist in all complex biological systems. These systems are involved at different levels, from cells to populations, and perhaps in species involving both mitotic and meiotic inheritances. At present most of this work focuses on molecular mechanisms, and few authors have attempted to investigate their evolutionary impact [143,144,154,155].

Epigenetic marks affect genome expression and genotype-phenotype relationships in general. This was recently discussed by Johannes et al. [155] in terms of quantitative genetics. As has been shown in plants [156], epigenetic modifications can be driven by environmental changes or stress. In general, stress can be responsible for modifying the epigenome and/or the selection of epialleles, leading to changes in the expression profile of gene(s). Hence the influence of the environment on the phenotype may be mediated by the epigenome.

In terms of evolution, the epigenetic status of the cells is important only if it affects the next generation. Initially most epigenetic modifications were thought to be only mitotically transmitted, but it is becoming increasingly clear that transgenerational transmission does occur, as recently reviewed by Jablonka and Raz [157]. Several examples of epigenetic inheritance involve transposable elements [67,124,158,159]. In Drosophila, hybrid dysgenesis involving *P*, *I*, and *Penelope *elements can be explained by transmission of small RNAs [121,160,161]. The evolutionary impact of such a feature is obvious, and several scenarios have been recently proposed and discussed [143,144,155]. Indeed, the description of epigenetic variation among individuals in population, and more importantly, the fact that epialleles can be selected, could become a corner-stone in explaining many evolutionary phenomena. In such a context, as Jablonka and Lamb [154] point out, the epigenetic phenomenon can be considered as a transient state before fixation occurs by genetic mutation(s).

### For the genome, TEs carry useful sequences and functions that can be exploited

**Figure 2:**
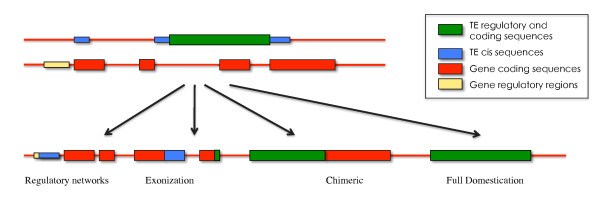
**The different levels of TE utilization by the genome**. TE sequences (green and blue) may contribute to gene regulatory regions (yellow, thin rectangles) or coding sequences (large rectangles). Small portions or almost entire elements can be exapted, which can result in new regulations or new genes.

Data accumulated over several years have indicated that the contribution of TEs to the evolution and function of host genes is far from negligible. The direct participation of TEs in genome functional evolution can occur in different ways (Figure 2). First they can carry sequences into regulating, coding, or intronic regions. These sequences may trigger useful functional changes (expression pattern, alternative splicing, transcription initiation and termination) as a result of the presence of particular motifs or their physico-chemical properties [see [162] as a recent example]. Second, they can provide a function normally encoded by the element, which is then recruited to implement a cellular function. In this case, either an entire domain or the full protein is recruited, i.e. domesticated by the genome. The molecular domestication of transposable elements has long been known to occur, even if the role of the domesticated copy in the cell is not always obvious [163,164]. It concerns both classes of TEs. The roles assumed by TEs in the cell are far from anecdotal, and can lead to important evolutionary innovation.

There are several criteria that may indicate that a domestication event has occurred: the loss of mobility, presence at only one locus, fixation in the population, presence of an intact open reading frame, presence at orthologous sites in several species, or traces of positive selection on these orthologous sequences [165]. Obviously, none of these criteria is sufficient in isolation, because each of them occurs in a normal TE life cycle. For example, traces of selection are visible in some cases of suspected horizontal transfer [166,167]. Fixation of an immobile copy in one or several species can be achieved simply as a result of demographic history and genetic drift. When a TE family is on its way to being eliminated, it loses its members one by one, until only one copy remains.

#### 1 - Exploiting TE functions

The genome advantageously uses the TE-encoded functions for its own purposes. Two situations can be distinguished: the entire protein may be domesticated, or only one domain. In the latter case, a chimeric gene is often created.

Full domestication is the most extreme case, in which the entire coding region is used to carry out the new function. The best known examples include the Drosophila telomeric retroelements HetA and TART, which function as a telomerase to heal chromosome ends. Classical telomerase contains a reverse transcriptase domain, which indicates that retroelements and telomerases may have a common origin, but it is unclear whether an ancient retroelement gave birth to the telomerase, or on the contrary originated from the telomerase. In the latter case, a "U-turn" of retroelements reverting to their original telomerase function would have occurred in Drosophila [168]. The envelope gene of some endogenous retroviruses is also involved in domestications, in particular in human and other mammals in which it induces the cell fusion required for syncytiotrophoblast (placenta) formation [169,170]. Among the DNA transposons, the vertebrate V(D)J recombination is a clear example. In this case, the transposase (from a *Transib *element) performs the recombinations necessary for setting up the immune system [171]. RAG1 essentially functions like a transposase, and the same enzyme activities are recognized (endonuclease and transferase). CENP-B is a centromere binding protein present in eukaryotes, which is derived from the Class II family of *Tigger-pogo-Fot1 *element [172], probably as a result of independent convergent domestication events [173]. Among the increasingly numerous examples, the best known, reflected by the introduction of a new function, correspond to evolutionary novelties with a great impact.

As described in Volff [165], two steps are necessary for an entire element to be tamed. One of the problems with TEs is their ability to move, to amplify and to be lost. Stabilization by immobilization of the copy in the genome prevents its loss by non-reinsertion (for excising elements) or recombination (LTR retroelements), as well as its further amplification by transposition. This is usually done by loss of the *cis*-sequences indispensable for transposition (often element termini, such as LTRs or TIRs). In practice, element truncation has often occurred, probably because it is independent of transacting factors [165]. This step is not the most difficult to achieve because the truncation of TEs occurs rather frequently. Another solution is to lose the transposition activity completely. This may be regarded as an ineluctable fate of TEs, which are thought not to be subjected to intense purifying selection for their transposition ability. Stabilization is not enough, since immobilized elements must also provide a function, and this is not usually transposition. Hence, changes in the coding sequence must also occur that alter the ability of the protein to perform (retro)transposition while conferring a new function on the protein (or maintaining some of its existing functions in new context, *e.g. *a DNA binding domain).

In many other cases, the domestication involves only part of the TE protein. TE proteins usually encode a limited number of functions. Transposases are characterized by a DNA binding domain, and a domain with endonuclease, and by strand transfer activities. Retroelements usually encode gag proteins (with DNA Binding domain, and antigenic properties), reverse transcriptase, integrase/endonuclease, and envelope proteins present in plasma membranes. In some atypical elements, such as DIRS, Cryptons and some IS elements, the enzyme is not referred to as an integrase/transposase, but is an (S or Y)-recombinase, which integrates DNA in a different way, usually *via *a circular intermediate [174]. A domain is often domesticated through its fusion with cellular protein domains [175-177]. In this case, one of the activities carried by the TE protein is retained. For example, DNA binding domains are frequently derived from class II DNA binding domains [reviewed in [178]]. The proteins containing these domains are involved in a variety of pathways, and for example, are easily hijacked for transcription factor functions. The increasing number of examples reveals the wide diversity of functions in which domesticated domains are involved [reviewed in [179]]. Hence the genome appears to be rather good at using a few basic activities to generate numerous functions, in a variety of pathways.

#### 2 - Exploiting TE sequences

When encoded functions are not required, a genome can also exploit TEs by using their sequences for other purposes. TE sequences may have interesting properties, non-coding sequences containing fortuitous ORFs, binding sites for regulation by proteins, or just useful chemico-physical properties.

The large-scale use of non-coding TE sequences as coding sequences by genomes was first revealed in the human genome. Several authors reported a relatively high proportion of TE sequences in exons, suggesting the process of exonization is not marginal and that various different kinds of TE are involved [180]. Similarly, the implication of exonized TE in the generation of alternative splicing has been recognized [181], sometimes with subtle effects [182]. However, in most cases alternative splicing is not synonymous with an exaptation event and, in the case of primate Alu elements, may be subject to loss in some species, suggesting that the exaptation process takes time to occur [183,184]. More convincing evidence of exaptation comes from the analysis of the more ancient MIR elements, which are found in all mammals [184]. Most examples of exonization (TEs in coding regions) are derived from analyses of mammalian genomes, in which TEs are frequently found within genes (introns). In other metazoans or eukaryotes, the phenomenon of exonization and alternative splicing appears to be less prominent. Lipatov et al. [185] found that chimeric TE-gene RNAs were rather rare in Drosophila, a fact explained as probably being a consequence of the deleterious effect of the TE insertions. In plants, alternative splicing and TE-mediated alternative splicing appears to be less frequent [186], but there are several examples of expressed chimeric genes derived from TEs that carry gene host fragments, such as PACK-MULEs [187]. Exonization has been shown to be more frequent in duplicated genes, which is consistent with the neofunctionalization theory [188]. It should be noted that gene duplication could also result from TE-mediated recombination [189].

TEs are also involved in the evolution of genome functions through their wide use as regulatory sequences [178]. Besides the numerous known examples of gene regulation through TEs sequences [[30,190], see also [191] for a recent example], a more general role in gene regulation was suspected after the discovery that some TEs (notably MITEs) tend to be located in the vicinity of genes [192,193], and that various regulatory motifs can be detected in some TEs [[194,195] for examples]. In the genomic era, comparative transcriptomics have made it possible to demonstrate the involvement of TEs in gene regulation variations, directly or through epigenetics [137,196]. Moreover, in mammals, studies of promoter regions, and transcription factor binding sites (TFBS) have revealed that a large proportion of sites originate from TE sequences [76,197]. Hence, the ability of TEs to amplify provides an easy way to modulate entire regulatory networks [178]. Such data supports the old hypothesis that TEs play a major role in regulation, and thus in evolution [46,198,199].

While the first examples of TE domestication and cooptation to be discovered appeared to be exceptional (although of prime importance with regard to function), more recent findings prove that this is in fact a recurrent phenomenon in genome history. From the beginning, genomes have regularly fed on TEs.

### Parasitic TEs and host fitness

At first sight, most of the DNA changes described above might have some deleterious consequences for the cell and for the organism. And indeed, in a worst case scenario, inactivating genes or rearranging chromosomes can have immediately lethal consequences. In other cases, the alteration of genes or their rearrangement may be less harmful (only slightly deleterious). However it looks as if a TE insertion usually has no dramatic impact if it occurs in dispensable/non-genic DNA for example (for instance in other TEs), which often corresponds to most of the genome. In rare cases, an insertion can even have beneficial effect, and so will ultimately become fixed.

However, transposable elements have tended to be known solely for their harmful mutagenic effects, which once raised the question of how they manage to survive despite natural selection. This implied that a genome with high fitness would be one with few TEs. But in fact this is rarely the case. First of all, we have to remember that transposable elements are the archetype of selfishness. Their only *raison d'être *is to amplify and perpetuate themselves in the genome. Encoding the ability to self-propagate within the genome is a simple but very powerful aspect of their selfishness. The "selfish genes" of Dawkins work in a much more complicated manner to propagate themselves in the population by exploiting sophisticated organismal "survival machines". When faced by threatening natural selection, it is far easier for a TE to duplicate itself than for a gene to do so. Second, when the genetic burden caused by TEs becomes too great, individuals or an entire population may become extinct. This may explain why many TEs are only found in moderate numbers of copies. Third, most TE insertions are in themselves probably neutral, as are most mutations, with some deleterious insertions that are ultimately eliminated, and occasional beneficial insertions that are eventually fixed. So, on average, the fitness cost of carrying TEs may be relatively limited.

So far we have only glimpsed the potential enormous positive impact of TEs on long-term genome evolution. However, this long-term benefit cannot be set against the short-term deleterious effects. Such a consideration resembles the sex paradox, where the benefits of sex (which generates genetic diversity) are visible in the long-term, but cannot offset the short-term, two-fold cost of sex compared to asexuality [200]. In both cases, the discrepancy in time scale is reinforced by a difference between the levels at which the effects act, at the individual level for short-term effect, and at the population or species level for long-term effect.

Genome-TE interactions are often viewed as an arms race in which each opponent successively devises fresh tricks to overcome the opponent's latest displays, resulting in tight co-evolution. TEs are genomic parasites, subjected to the fire of natural selection that may act directly on any insertion, or indirectly by favoring on the one hand a genome with good defenses, and on the other hand TEs that are able to tame themselves. The ultimate weapon developed by the genome is the impressive epigenetic defense system that does not destroy TEs but efficiently silences them, as can be observed in Arabidopsis [67]. Arms race is visible in the rapidity with which proteins involved in the defense system evolve, but in contrast, the rate of evolution of an element is difficult to infer. However the huge diversity of TEs suggests that this arms race does indeed exist. Of course, each time a TE escapes from epigenetic control, amplification bursts can occur (and indeed do occur from time to time). TEs can also escape control as a result from their ability to colonize new hosts after horizontal transfers.

## Evolution of the TEs embedded in the genome

While the impact of TEs on the genome has been the focus of many studies, only a few have looked at the impact of the genomic environment on TE evolution. The dynamics of TEs are usually inferred from population genetics, and the use of analytical or simulation models, and there are few experimental studies or biological data [201]. An emerging approach is exploring this issue from an ecological point of view, looking at TEs as individuals living in the genome [202]. Finally, comparative genomics may also be used to help us to understand the evolution and dynamics of TEs.

### TE dynamics are influenced by several parameters

#### 1 - The accepted hypothesis (transposition is balanced by selection or self-regulation)

It is widely accepted that the evolution and dynamics of TEs are governed by a balance between transposition and selection [203]. It is assumed that transposable elements are slightly deleterious and decrease host fitness, and so tend to be eliminated, whereas the transposition process tends to increase the genomic copy number, in a purely selfish manner. Different models suggest that TE purifying selection result from deleterious insertions within genes, from deleterious ectopic exchanges responsible for genomic rearrangements [204-206], or from a poisoning effect of TE activity [203,207]. Selfishness derives from the fact that TEs are able to replicate more rapidly than the host genome [38,91]. Although both forces clearly do apply, there is no need to reach this equilibrium to explain the persistence of TE over very long periods of time [208]. First, sudden changes disrupting the equilibrium are recurrently observed (transposition bursts, variable deleterious effects). Secondly, other non-adaptive forces must also be considered (see below). Third, TEs have evolved as thousands of different families, each with its own history. The extant TE diversity is probably only a small part of the total historical diversity, and the persistence of some TEs and the disappearance of some others are in themselves non-adaptive and rely, at least in part, on stochastic mechanisms. This means that the evolutionary history of TEs can be explained without necessarily involving long-term, stable copy number equilibrium.

#### 2 - Effects of population size, host demographic history, and genetic drift

The effective population size (Ne) is described as having an important impact on the evolution of genome architecture [209,210], including TE diversity and polymorphism. According to Lynch and Conery's hypothesis, selection is less effective at purging TEs in small populations, because genetic drift is stronger as the effective population size Ne decreases [210]. Again, the model assumes that TEs have a slightly deleterious effect, which is confirmed by several analyses, including that of Pasyukova et al. [211] estimating that on average a TE insertion decreases the fitness of an individual by 0.4%. A recent population genetics study of several TEs in plant populations of which the demographic history is known suggested that TEs diversity is influenced by demographic factors such as bottlenecks and population size fluctuations [212]. Another example comes from the invasive *Drosophila simulans *species, in which the level of the *mariner *element activity increased as the migration distance increased, probably as a result of repetitive bottlenecks [213]. However, simulation studies suggest that genetic drift is a significant force in eliminating TEs from small populations [208].

#### 3 - Effects of recombination and of reproductive mode

The invasive properties of TEs include their abilities to multiply within one genome and to spread within the population. This is of prime importance for newly arrived TEs, which are initially present in just a few copies in a few individuals, and that have to invade both the genome and the population, but also for TEs that are already established in a species. Hence the reproductive mode is an important factor influencing TE dynamics.

TEs have been described as sexually-transmitted parasites [214]. Indeed the model predicts the inability of TEs to invade species in the absence of sex: an element arriving in the genome of an asexual individual would be able to invade this genome, but not to colonize genomes of other lineages during zygote formation. Moreover, the loss of sexuality of a species already containing TEs may lead to the progressive loss of the TEs, or at least of TE activity, because TE proliferation would cause extinction of the lineage due to detrimental effects [215]. At most, copy-number equilibrium may be attained under certain specific conditions (infinite population and no excision at all). However, in small populations, the TE load leads to extinction, while in larger populations genomes could get rid of the TEs [216].

All these predictions appear to be difficult to demonstrate in nature. Among eukaryotes, the bdelloid rotifers correspond to well-established, ancient, asexual organisms. However, the search for TEs in these species has led to the discovery of several families of Class-I and -II elements [217,218]. The hypothesis suggested is that the presence of TEs results from repeated horizontal transfers [219]. Moreover, TEs appear to be severely confined to specific chromosomal compartments [218]. Ancient asexual haploids are probably best represented by prokaryotes. When compared to eukaryotes, overall they carry a smaller load of mobile elements, which may be explained by enhanced selection due to haploidy and small-sized genomes. However, most prokaryotes nevertheless contain IS elements. In addition to any benefits they may carry (antibiotic resistance, genome plasticity), their persistence could result from a rapid turnover, with frequent horizontal transfers offsetting rapid losses through selection [220].

Asexuality represents the most extreme situation, but nature is full of species with sexual behavior that is somewhere between full asexuality and obligate out-crossing sexuality, notably if we consider their recombination ability. Hence, differences in the ability TEs to invade or to maintain itself in a population are also to be expected between selfing or out-crossing sexual species [221].

Reduced genetic exchanges (as in selfing populations) leads to greater variation in TE copy number, and thus to stronger natural selection forces [214]. When the effect of selfing was analyzed in different selection models, contrasting results were observed, with negative correlations between the copy number equilibrium and selfing rate in the transposon insertion model (heterozygous or homozygous) [222], but positive correlations in the ectopic exchange model [206,223]. Under self-fertilization (autogamy), genetic exchange is limited and ultimately results in a high level of homozygosity. Langley et al [205] suggested that TEs could accumulate in regions with low levels of recombination. This is observed for the heterochromatic regions (pericentromeric, telomeric). At the population level, effective recombination (including deleterious ectopic recombination) is thought to be reduced in highly homozygous (highly selfing) species. Charlesworth et al. [224,225] suggest that more abundant TEs may therefore be allowed in selfing species, a hypothesis that is still controversial [see also [135,226]]. Furthermore, as proposed by Wright and Schoen [206], recessive mutations caused by TEs have more impact on homozygous genomes, leading to stronger selection against TEs. Therefore contradictory findings may be expected, depending on the relative importance of deleterious effects of insertions or ectopic exchange on the overall host fitness [223].

Simulations confirm that in the insertion model, the chance that a TE will invade a genome is drastically reduced when selfing increases, because of the reduced genetic exchange and reduced effective population size. Moreover, under conditions in which molecular domestication events can occur, such events appear to be delayed. Finally, when the adaptive insertion rate is low, TE activity displays a cyclical pattern, with a higher periodicity than under out-crossing conditions (TS Boutin, A Le Rouzic and P Capy, unpublished data). Comparative studies have also been performed in real selfing and out-crossing species. In nematodes, as in Arabidopsis, it was found than insertions were less polymorphic and segregated at higher frequencies in selfing species, which would be compatible with a relaxed selection in selfing species, population size reduction or reduced transposition rate [227,228].

### The TE lifecycle

The emergence of TEs in a naive genome may have two origins. The first, and perhaps the most frequent origin, is the horizontal transmission (HT) of an active copy into the germ line. This phenomenon is frequent in prokaryotes, and the mechanisms of transfer are known (conjugation, transformation, and transfection). In eukaryotes, such transfers seem to occur far less frequently, and their mechanisms remain unknown. It is quite possible that one or several intermediates, including bacteria, viruses, or parasites, could be required [229]. However, whatever the mechanism, the TEs must reach the germline.

Comparisons of HT frequency show clearly that significant differences exist between the main superfamilies. Recently, Loreto et al. [229] estimated that among the 98 HTs described in *Drosophila*, 51% involve DNA transposons, 44% LTR retrotranposons and 5% non-LTR retrotransposons. Quantitative estimations cannot be provided for other species, but several cases of HT have been reported in mammals and tetrapods [230], in bdelloid rotifers [231], and in plants [232].

The alternative hypothesis of TE origin is the *de novo *emergence or re-emergence of autonomous sequences as a result of recombination between inactive copies. While there is less supporting evidence for this, it has been demonstrated that ectopic recombination between different copies of the same family or copies from different subfamilies can occur. For instance, it has been shown that some of the TEs described in yeast as *Ty1/2 *elements are in fact hybrids between *Ty1 *and *Ty2 *[233]. More recently, Sharma et al. [234] reported new elements resulting from repeated recombinations that may occur during the hybridization of sympatric species and polyploidization. Similarly, Marco and Marin [235] showed the emergence of a new *Athila *lineage as a result of recombination between distantly-related copies. In all these cases, this is not a *de novo *emergence; it is rather a re-emergence of autonomous copies from non-autonomous or dead copies.

As soon as a new element appears in a naive genome, it has to face a new challenge since there is generally a single copy, in a single individual, in a single population. To avoid being lost, this copy must invade the population and the genome. The transposition rate estimated from several natural populations, laboratory strains and for several types of elements is about 10^-4 ^transpositions/copy/generation. If we apply this rate to a newly arriving copy, this copy would almost systematically be lost. Therefore, two scenarios for a successful invasion have been proposed. First, either a high rate of new elements arriving by HT or recombination, or a high transposition rate of the initial copy i.e. close to 10^-1 ^or 1, according to the model prediction [236]. Of course, such a transposition rate cannot be maintained for long without risk to the population. Therefore, regulation of the transposition rate can be expected to occur rapidly. This could result from self-regulation by the TE or host regulation [203].

After the successful initial invasion of the genome and of the population, it becomes difficult to lose an element. Thus, it is important to follow the TE dynamics both in the genome and in the species. In most of the models published in the 1980s and 1990s, it was assumed that the copy number of an element had to reach an equilibrium (see [201]). However, most of these models failed to take the impact of mutation on TE activity into consideration. When this effect was included in the model, it could be shown that it is almost impossible to reach a long-term equilibrium, and several dynamic outcomes can be observed, including the loss of the active or *trans*-mobilizable copies, or the domestication of a copy.

### TE competition and the ecology of the genome

With the exception of a few species, a genome does not normally contain only one type of TE. For a given family, several types of copies with differing levels of activity can be detected, including inactive copies. This is clearly demonstrated by analyses of a large number of genomes involved in sequencing projects. Since this situation is observed for almost all TEs, several questions arise: Is there any competition between different families, or between different types within the same family? Can an equilibrium resembling an Evolutionary Stable Strategy (ESS) be reached by these TEs in a genome? Can we apply models of population biology to the dynamics of TEs in a genome?

**Figure 3:**
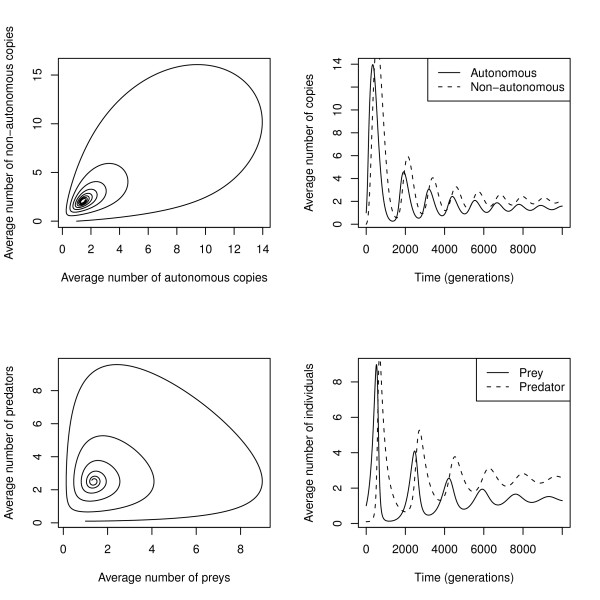
**Illustration of the similarities between the theoretical dynamics of autonomous/non-autonomous TE copies (top row) and prey-predator models (bottom row)**. The transposition model is described in Le Rouzic & Capy 2006. See Additional File 1 for more details about equation models.

In the last few years, it has been assumed that the genome can be viewed as an ecosystem in which TE copies are considered as individual members of a species [202,237,238]. In such an analogy, autonomous and non-autonomous copies of the same family are competing entities rather than belonging to the same "species". In any case, the resources are produced by autonomous or truncated copies that have kept an intact ORF. These resources correspond to the transposition machinery like the transposase for the Class II elements, and can be used both by autonomous copies and by *trans*-mobilizable non-autonomous ones. Simulations and analytic models both provided TE cyclic dynamics due to the competition between active and non-autonomous copies, which are similar to the prey-predator dynamics described by Lotka and Volterra in population biology [[238], see Figure 3 and Additional file 1]. In such a context, non-autonomous copies can be viewed as parasites of autonomous copies, providing a nice illustration of how a genome can be viewed as an ecosystem. This cyclical pattern may be disrupted by changing any of the parameters of the system, leading occasionally to the loss of one or other of the elements. Hence TE interactions within a family should probably not be considered to constitute an ESS (Evolutionary Stable Strategy). Furthermore, it must be stressed that transposition bursts may occur [239-242], and that these sometimes reflect perturbations that can lead to long-term changes in TE content.

In addition, several families may coexist in a single genome. TE interactions have been poorly studied in cases where *trans*-family mobilization is not possible. However, it looks likely that if the genome is considered as an ecosystem, its "biotic capacity" is probably restricted. In other words, the expansion of a given family could have an impact on the dynamics of another family, reflecting a struggle for survival between TE families similar to that which occurs between species sharing the same ecological niche.

## Concluding remarks

The genomic and post-genomic eras have unraveled the importance of TEs in genome evolution. The early suspicions, including McClintock's predictions, turn out to be true: TEs do indeed play a significant role in gene evolution, at least in some species, in gene regulation, and in the genomic response to stress. A number of isolated examples are now seen to reflect a general rule. At the time of McClintock's work, TEs, which she called controlling elements, were considered to be part of the genome. Today, they are perceived as being independent of the host carrying them, although more intricate relationships have been revealed: a large fraction of the genome is now known to be implicated, molecular domestication has occurred, TEs are directly implicated in the formation of regulatory and coding sequences, play direct and indirect roles in key cell processes, and have responsibility for lateral gene transfers, at least in prokaryotes.

During the last decade, the numbers of known TE families has grown, as has the number of examples of TE domestication, and the rhythm of publications on TEs has quickened.

Methods for identifying transposons have changed considerably over this time. Fortuitously discovered to begin with, TEs were then searched for using various methods, including transposon trapping, and are now mainly identified as repeated sequences with particular features within sequenced genomes, or through protein homology, with the help of various and numerous programs and databases. Still more recently, new superfamilies, and completely new types of mobile element have been discovered [18,19]. The explosion of metagenomic and genomic sequences make the identification of all TEs a costly and challenging task. The use of bioinformatics, with sophisticated and efficient detection programs is a crucial element in attempting to provide a comprehensive survey of TE diversity and evolution.

Technical progress now makes it possible to trace the history and dynamics of TEs within a genome, and grasp both the influence of TEs on genome specificity and the influence of the genome on TE evolution. The dichotomic view of TEs as either parasites or necessary beneficial entities is resolving towards a unified view, in which these are two aspects of the same process, which merge to form a continuum [39]. TEs and genomes have probably been in constant contact since life began, and this cohabitation has had repercussions on the evolution of both partners. Hence it is not surprising to discover that TEs have beneficial effects since parasitic elements and genome sequences have mixed for so long.

However, some of the old questions remain unanswered, while new questions have arisen: the horizontal transfer of TEs appears to be a major step in TE evolution and propagation. In eukaryotes, this phenomenon is rare, and its mechanism (or vector) still unknown. Continued investigations in this field and careful analysis of the findings must be pursued.

The epigenetic component of genome functionality has been the focus of intense interest in the biology community in recent last years, and this has replaced TEs centre-stage. However, TE-genome relationships within the epigenetic dimension are far from having been deciphered, and still require intense research.

TEs are parasitic by definition and like parasites, TE expansion depends on interactions with the host, i.e. the genome. This part of the TE biology remains to be explored, since the vision of TEs as competing individuals, or species, within their ecological niche, the genome, or struggling with their own parasites (non-autonomous elements) is rather new. Addressing this poorly investigated aspect will be facilitated by the availability of the genomes of several individuals per species within few years.

## Abbreviations

TE: transposable element; RT: reverse transcriptase; LTR: long terminal repeat; LINE: long interspersed transposable element; PLE: Penelope-like element; SINE: short interspersed transposable element; TIR: terminal inverted repeat; IS: insertion sequence; MITE: miniature inverted transposable element; ICE: integrative and conjugative element; Tn: composite transposon; LGT: lateral gene transfer: HT: horizontal transfer/transmission; SNP: single nucleotide polymorphism; S/MAR: scaffold/matrix attachment region; MULE: Mutator-like element; RIP: repeat-induced point mutation; RNAi: RNA interference; TGS: transcriptional gene silencing; PTGS: post-transcriptional gene silencing; ncRNA: non-coding RNA; siRNA: small interfering RNA; miRNA: microRNA; piRNA: PIWI-interacting RNA; rasiRNA: repeat-associated small interfering RNA; dsRNA: double-stranded RNA; VRS: virus RNAi silencing; TFBS: transcription factor binding site; CRISPR: clustered regularly interspaced short palindromic repeats; R/M: restriction/modification; RdRP: RNA dependent RNA polymerase; ESS: Evolutionary Stable Strategy

## Reviewers' comments

### Reviewer 1

Jerzy Jurka, Genetic Information Research Institute

This reviewer provided no comments for publication

### Reviewer 2

Jürgen Brosius, University of Muenster

This is a review on the impact of TEs on the evolution of genomes and genes including their regulation and epigenetic phenomena. Unlike many previous reviews, parts of the present one explore the (potential) significance of TEs from different angles and hence make it a worthwhile read. At the same time, the manuscript still carries the burden of past and present misunderstandings or ambiguities concerning TEs.

1) One of the difficulties to write a general review on all classes of TEs, is the fact that they are very different from each other, especially when comparing DNA transposons (class II) that usually operate in the cut and paste mode and class I TEs that operate in a copy and paste mode via an RNA transcript. Although this is discussed beginning on page 7, perhaps for the reader it would be easier to mention it at the onset. As an aside, few class I elements are transposABLE elements as, after integration into the genome most copies are not able to produce additional copies because they are dead on arrival. Especially, SINEs are rarely being transcribed because they lack the necessary flanking regions for autonomous transcription while LINEs are mostly truncated. An interesting exception is a small nucleolar RNA (snoRNA)-derived class of SINEs in Platypus that often maintains the ability to be co-expressed and processed into distinct RNAs when retroposed into introns of RNA polymerase II genes [1]. Nevertheless, in most cases a better description would be transposED elements for class I TEs.

**Authors' response: ***In this manuscript, we used a classical terminology to describe transposable elements. "Transposable elements" are generally defined as sequences able to promote their own mobility and/or duplication in genomes, but in practice this definition largely extends to all TE-derived sequences, even if they have lost this autonomy. Consequently, non-autonomous copies (such as SINEs or MITEs) are generally considered as transposable elements (even if they are phylogenetically unrelated to the corresponding autonomous element), as well as totally inactive TE-derived pseudogenes, even if these are not actually "transposable". On the opposite, retroprocessed sequences that are not repeated (but just happened to be reverse-transcribed accidentally) are not considered as transposable (perhaps "transposed" here would fit). Although non-homologous, "transposable elements" and their derived sequences thus constitute an unambiguous group of sequences, characterized by their distribution in genomes (they are the middle repetitive fraction of the genome DNA), their evolutionary properties, and their "ability to transpose", where "transpose" stands for both "copy and paste" and "cut and paste" mechanisms*.

**Reviewer's response: **Good point! Use "classical" for a term that is imprecise and not quite correct and you are off the hook. An admittedly extreme would be the "classical view" of the sun revolving around the earth. Reviews often address readers outside the field. Imprecise terminology leads to misconceptions that are difficult to purge. Hopefully readers will get as far as this section. Were I not familiar with this research topic, I would have been confused about much of the content concerning class I elements including the following statement from the background section (despite the attempt for clarification in the last sentence):

"**1 - TEs are a major factor in evolution because they are an important source of variability**. Mutations caused by TEs are diverse, ranging from small-scale nucleotide changes to large chromosome rearrangements, including epigenetic modifications. Although TEs are mobile, the nucleotide (or epigenetic) changes resulting from their transposition can persist, being transmitted through generations and through populations."

With respect to class I TEs, it is not the DNA that is moving but the RNA. RNA is transcribed from the DNA and then reverse transcribed and integrated. Neither master copy(ies) from which the RNA is originating nor the numerous integrated cDNAs do not move once integrated into their respective loci (except by genomic rearrangement as for any piece of DNA). If these class I TEs weren't absolutely sedentary, they could not be used as reliable phylogenetic markers as mentioned in this review under the heading "From mutations and (epi)-genetic variation to genetic novelty and adaptation". Actually, the first publication on the phylogenetic potential of retroposed elements (Alus) came from A. Dugaiczyk's laboratory: Ryan, S. C., and A. Dugaiczyk. 1989. Newly arisen DNA repeats in primate phylogeny. Proceedings of the National Academy of Sciences USA 86:9360-9364. N. Okada's laboratory perfected the approach to solve many interesting phylogenetic questions. This effort is summarized in the following review: Shedlock, A., K. Takahashi, and N. Okada. 2004. SINEs of speciation: tracking lineages with retroposons. Trends in Ecology and Evolution 19:545-553. Perhaps, part of the discrepancy stems from the DNA-centric majority view versus my RNA- centric view (see also comments 3 and 11).

Another point: I do not see how TE insertions cause small-scale nucleotide changes. Those changes are at least as large as the TE, i.e., at least 70 nt plus direct repeats.

***Authors' response: ****If one considers mobility or transposability as the ability to excise, then we agree that Class I are neither mobile nor transposable. But if one considers mobility or transposability as the ability for a DNA segment to be inserted at a new position, then, Class I element are TEs. It seems that this last definition is more universally used. On what, in our opinion, should be considered as TEs, see comment 11*.

*Small-scale nucleotide changes occur when Class II elements excise. Usually the initial sequence is not exactly restored after double strand break repair. This is referred to as the excision footprint (added in the text)*.

2) The title of the manuscript already contains two terms that require qualification. First of all, non-autonomous TEs are not necessarily selfish. In theory, any RNA (including messenger RNAs) can be retroposed [2]. Some, however, are reverse transcribed and retroposed in a highly efficient manner by the machinery of autonomous TEs (e.g., LINEs) and hence give rise to thousands, up to a million copies in a genome. Of course, one could argue that some of the frequently retroposed RNAs do not have cellular functions any longer, but evolved structures that tricks the machinery of autonomous TEs into using them as templates [1]. Even though the RNAs might have lost (original) function, genes encoding such RNAs survive, not necessarily at its original genomic locus, but fortuitously due to the sheer number of copies generated. As a consequence, a few of them are bound to integrate into a genomic locus that permits autonomous transcription [3]. This is probably the case with Alu elements that survived in some form or another from the beginning of mammalian radiation up to now. Such a mode of persistence is documented, for example, by the presence of Alu subfamilies that were active at different times in primates. Of course, one cannot rule out a cellular function of some Alu RNAs at this juncture. The second problematic term is "architects" which implies foresight and planning. Perhaps, the term "agents" would be less ambiguous. In a similar vein, perhaps one should stay clear of the term "create" or similar (used elsewhere in the text).

**Authors' response: ***In theory, there is indeed a stage where a non-selfish sequence (as e.g. the ancestor of Alu elements) starts, for some reason, to be amplified by an autonomous copy. However, genome-level selection will take place very rapidly, and among all amplified copies, the one that have a slight "superparasitism" advantage will become more frequent than the others, and the original ancestors will be outnumbered. Since there is no reason why the mutations favoring transposition would maintain the original cellular function, the probability for a sequence to be both selfish-DNA and "altruistic DNA" at the same time remains infinitesimal. The same infinitesimal (at the evolutionary scale) transition period exists when a functional copy inserts in a site where it brings some selective advantage, the copy being both potentially selfish and useful. A similar unstable stage probably also exists when species evolve from e.g. parasitism to symbiosis, but does not preclude an operational classification between two non-exclusive categories. As in remark #1, the issue is probably linked to the fact that "selfish", in the same way as "transposable", generally also qualifies derived sequences that are not by themselves "selfish" or "transposable", but exist because their direct ancestors were selfish and/or transposable*.

*Although the reviewer's remark about the use of the term "architect" is formally exact, we note that similar stylistic effects are common in the literature (Mattick (2001) "Non-coding RNAs: the architects of eukaryotic complexity" EMBO reports 2, 11, 986-991), and our feeling was that our "selfish architects" could not be understood in a different way than e.g. Dawkins' blind watchmaker. Potentially misleading occurrences of "create" were removed from the text, and we believe that this comment published along with the article will prevent misinterpretation of the title*.

**Reviewer's response: **Concerning the infinitesimal probability for a sequence to continue to be both selfish-DNA and "altruistic DNA" at the same time, BC1 RNA is a counter example. It arose in a common ancestor of rodents via retroposition of a tRNA, has a function in the central nervous system and is the master copy of thousands of ID repetitive elements generated over long time periods. However, as the authors stated above, a few rare integrated copies that happened not to be transcriptionally silent, became master copies of additional sub-families of ID repeats [reviewed in ref. [2], given at the end of this section]. Once more, for class I TEs, it would be the RNA that is selfish, not the bookkeeping DNA [7], just as the RNA of an RNA virus would be selfish and not the integrated genomic DNA copy. On the other hand, DNA transposons (class II) might be considered selfish DNA.

**Authors' response: ***This is indeed a nice counter example. Retroposition ability and cellular function may be both present because the gene is in a transitional stage before retrotransposition ability be lost, or because both reside on the same sequence in the gene (the non-tRNA part)*.

3) For most investigators, evolutionary considerations begin with the last common ancestor (LUCA) with RNA, protein and DNA already in place. A look at the RNA world and major evolutionary transitions [4], especially those from the RNP world to modern cells with DNA as bookkeeper, provides some scenarios to questions [5-8] such as: "Are we able to understand why they [TEs] are here, and why they are still here?". This also should qualify the statement, "the Central Dogma could not be questionable". See ref. [2], Figure 3.

**Authors' response: ***the reference to the Central Dogma was indeed unnecessary here, and we have reformulated this sentence. In order to address a remark from reviewer 1, most open questions were reformulated, so that we could not directly refer here to the origin of DNA*.

4) "TEs possess two main characteristics that distinguish them from classical genes...". One should remind the reader that some TEs are not genes. LINEs and LTR elements are more like small operons and thus harbour at least two genes. Furthermore, most SINEs or mRNA-derived retrocopies are not true genes but inactive pseudogenes (SINEs with extremely high copy numbers).

**Authors' response: ***Of course, we wanted to refer to non-TE sequences. This sentence was changed into "... from other genomic components"*.

5) The sentence " the core of Darwin's theory was never really questioned" needs qualification. Perhaps, the authors mean that it was never questioned in the scientific community. Even that would be inaccurate, see refs. [9-12].

**Authors' response: ***This sentence was indeed misleading, we meant that it was never successfully challenged. This was fixed in the revision*.

6) There are earlier references (in addition to refs. [34,35] concerning "TE as major actors of diversity" [13-15].

**Authors' response: ***The Kidwell and Lisch (1997) reference seems well adapted here since they review the effects of all classes of TEs in both animals and plants. The second reference illustrates through several examples the involvement of epigenetics in TE-induced phenotypic variations*.

7) "homologues of the three proteins involved in RNAi (ARG family, DICER and RdRP) can be found in all supergroups"

What is meant by "supergroups" major clades perhaps?

**Authors' response: ***Eukaryotes are divided in 6 clades called supergroups (Rhizaria, Chromalveolates, Archaeplastidae, Opisthokonts. Amoebozoa and Excavates. The very same term is used in the cited reference, and elsewhere to refer to these 6 clades*.

8) When discussing the CRISPR elements, it should be mentioned that the small RNAs were acquired from invaders, such as phages. The acquisition of these elements even resembles something akin to Lamarckism [16].

**Authors' response: ***We gave a little more details on these very interesting CRISPR elements. Contrary to Koonin and Wolf 2009, we are however a bit reluctant to qualify this process as "Lamarckism" (Lamarck's theory, which was a general framework to explain evolution, cannot be validated by rare observations in which Darwinian evolution has led to a system superficially similar to Lamarck's wrong model of evolutionary change)*.

9) "First they [TEs] can bring sequences in regulating, coding or intronic regions. Those sequences may trigger useful functional changes (expression pattern, alternative splicing, transcription initiation and termination), by the presence of particular motifs or their physico-chemical properties [see [144] as a recent example]. Second, they can bring coding sequences, which modify the initial sequence and create new genes. Concerning "coding sequences" I do not see much difference between "first" and "second", once you bring TEs into coding sequences, they usually have to be coding or they would destroy the ORF.

**Authors' response: ***Indeed, the second point was already included in the first, and has been removed*.

10) "The full domestication is the most extreme case in which the totality of the coding region is used to ensure the new function." True, but one should find smaller contributions of fragments of TE-derived genes (this is mentioned only in the legend to Figure 2). For example as novel (alternative) exons oder contributing new termini to existing proteins, just as mRNA-derived retrocopies do [17].

**Authors' response: ***This is what we meant by "extreme case". We refer to the less extreme cases two paragraphs later, "In numerous other cases, the domestication concerns only a part of the TE protein [...]". Here, the domain function is exapted. Smaller contributions are mentioned in the next part, which depicts exaptation of TE sequences (and not TE protein function). In some cases, exapted sequences become part of a coding region*.

11) "While first examples of TE domestication and cooptation appeared as the exception (although of prime importance in regard to the function), the recent and numerous data prove that this is actually a recurrent phenomenon in genome history. Since the beginning, genomes regularly feed on TEs," and "TE and genome have been in constant contact since probably the beginning of life and such promiscuity has had repercussions on the evolution on both partners." Genomes ARE transposed (RNA) elements [5-8,18].

**Authors' response: ***In this paragraph, we refer to transposable elements, and not to other sequences retroprocessed accidentally. We think that "transposition" is too specific to be applied to any kind of reverse-transcription event*.

**Reviewer's response: **There is not much difference between class I transposable elements (retroposons) and other retroprocessed sequences. Once more, the key to the difference lies in the properties of the RNA: some are more others less efficient templates for retroposition. Where do you draw the line: One hundred retrocopies of a tRNA are retropseudogenes and one thousand copies of a tRNA or tRNA-like RNA are SINEs?

**Authors' response: ***The copy number is clearly not the good criterion to decide whether a sequence is a TE or not. The property to be efficiently retroposed is crucial, and must not depend on the environment, meaning that basically, RNA produced from any intact retroposed copy must keep the ability to be reinserted*.

12) page 25, Exploiting TE sequences

A discussion about the persistence of exapted TEs in short evolutionary branches (gain and loss of exapted TEs e.g., in primates) [19] and long evolutionary branches (e.g., constitutive expression of exapted TEs in deep mammalian branches) [20] should be added.

**Authors' response: ***This discussion on the long-term persistence of domesticated sequences is indeed interesting, and is now mentioned in the manuscript. However, it is also important to consider that there is no strong evidence that TE-derived exons behave in a different way than new coding sequences from different origins, and that this could simply reflect the "average" fate of genetic novelties in genomes*.

**Reviewer's response: **Agreed, there should be no difference between TE-derived novel exons and those from anonymous genomic sequences [8], because even the latter are ancient TEs who are not discernible anymore, due to mutations over long time periods [6,18]. Actually, most if not all genomic DNA is TE-derived, which would return us to evolutionary transitions following the RNA and RNP-worlds [5,6].

13) Page 32, TE competition and ecology of the genome

For marsupials, Nilsson et al. could show an overlapping activity of RTE and LINE mobilized SINE elements along a single phylogenetic marsupial branch. The parallel activity of the two different retropositional systems was further supported by detecting frequent nested insertions of RTE in LINE mobilized elements and vice versa [21].

**Authors' response: ***There is indeed no doubt that several TE families can be active simultaneously in genomes. Reciprocal transpositions in inserted copies is a strong piece of evidence that this was the case in the marsupial lineage, and such a coexpression is regularly observed in modern insect species. The missing information, however, remains the degree of interaction between these families: do they use the same resources, do they fight the same regulation mechanisms? So far, it is not clear whether co-invading TE families are competitors, commensals, or mutualists*.

14) An additional earlier reference for the role of TE-derived genes in placenta formation should be cited [22].

**Authors' response: ***The literature has been updated*.

Reference:

[1] Schmitz J, Zemann A, Churakov G, Kuhl H, Grützner F, Reinhardt R, Brosius J Retroposed SNOfall--a mammalian-wide comparison of platypus snoRNAs. Genome Res 18:1005-10.

[2] Brosius J (1999) RNAs from all categories generate retrosequences that may be exapted as novel genes or regulatory elements. Gene 238:115-34.

[3] Brosius J (2003) The contribution of RNAs and retroposition to evolutionary novelties. Genetica 118:99-116.

[4] Szathmáry E, Smith JM (1995). The major evolutionary transitions. Nature 374: 227-32.

[5] Brosius J (2003) Gene duplication and other evolutionary strategies: from the RNA world to the future. J Struct Funct Genomics 3:1-17.

[6] Brosius J (1999) Transmutation of tRNA over time. Nat Genet 22:8-9.

[7] Brosius J (2005) Disparity, adaptation, exaptation, bookkeeping, and contingency at the genome level. Paleobiology 31(2 Suppl):1-16.

[8] Brosius J (2005) Echoes from the past--are we still in an RNP world? Cytogenetic Genome Res. 110: 8-24.

[9] Kellogg, Vernon L. (1907) Darwinism To-Day. A Discussion of Present-Day Criticism of the Darwinian Selection Theories, Together with a Brief Account of the Principal Other Proposed Auxiliary and Alternative Theories of Species-Forming" Henry Holt and Company, New York.

[10] Hull, David L. (1983) Darwin and his Critics. The Reception of Darwin's Theory of Evolution by the Scientific Community. University of Chicago Press, Chicago, ISBN 0-226-36046-6

[11] Mayr, Ernst (1991) One Long Argument. Charles Darwin and the Genesis of Modern Evolutionary Thought. Harvard Univiversity Press, Cambridge, ISBN 0-674-63905-7

[12] Woese CR (2004) A new biology for a new century. Microbiol Mol Biol Rev 68:173-86.

[13] Brosius J (2001) Retroposons--seeds of evolution. Science 251:753.

[14] Brosius J, Gould SJ (1992) On "genomenclature": a comprehensive (and respectful) taxonomy for pseudogenes and other "junk DNA". Proc Natl Acad Sci USA 89:10706-10.

[15] Brosius J, Tiedge H (1995) Reverse transcriptase: mediator of genomic plasticity. Virus Genes 11:163-79.

[16] Koonin EV, Wolf YI (2009) Is evolution Darwinian or/and Lamarckian? Biol Direct 4:42.

[17] Baertsch R, Diekhans M, Kent WJ, Haussler D, Brosius J (2008) Retrocopy contributions to the evolution of the human genome. BMC Genomics 9:466.

[18] Brosius J (2009) The fragmented gene. Ann NY Acad Sci 1178:186-93.

[19] Krull M, Brosius J, Schmitz J (2005) Alu-SINE exonization: en route to protein-coding function. Mol Biol Evol 22:1702-11.

[20] Krull M, Petrusma M, Makalowski W, Brosius J, Schmitz J (2007) Functional persistence of exonized mammalian-wide interspersed repeat elements (MIRs). Genome Res 17:1139-45.

[21] Nilsson MA, Churakov G, Sommer M, Van Tran N, Brosius J, Schmitz J (2010) Tracking marsupial evolution using archaic genomic retroposon insertions. PloS Biol 8:e1000436).

[22] Mi S, Lee X, Li X, Veldman GM, Finnerty H, Racie L, LaVallie E, Tang XY, Edouard P, Howes S, Keith JC Jr, McCoy JM. Syncytin is a captive retroviral envelope protein involved in human placental morphogenesis. Nature. 2000 Feb 17;403(6771):785-9.

### Reviewer 3

I. King Jordan, School of Biology, Georgia Institute of Technology

In this manuscript, Hua-Van et al. present a fairly extensive review of the interactions between transposable elements and their host genomes. The review emphasizes the numerous ways that transposable element derived sequences have influenced the structure, function and evolution of genomes and tries to reconcile these influences with classical (neo-)Darwinian evolutionary theory. The review is distinguished by the fact that it deals with two perspectives on transposable elements that are usually treated separately: the impact of the transposable elements on their host genomes and the function and evolution of the elements themselves. This paper makes a nice contribution to the field of transposable element biology and also fits well with the recent series of papers that Biology Direct has published dealing with current perspectives on Darwinian evolutionary theory.

Much of what is covered in this review has been treated elsewhere previously. Nevertheless, it is both timely and useful to have much of this material presented together in an evolutionary framework. Some of the newest and most relevant material is on the relationship between transposable elements, RNA interference and epigenetic phenomena. From my admittedly biased perspective, this represents the single most important contribution of this review. But this is an area of investigation that is changing rapidly, and I would urge the authors to consult some of the most recent literature on transposable elements and epigenetics to deepen this part of the manuscript. With all apologies for being self-serving, our own lab has recently published a couple of reviews on these topics: (Jordan IK and Miller WJ 2009 Genome defense against transposable elements and the origins of regulatory RNA in Genome Dynamics and Stability Lankenau and Volff (Eds) 4: 77-94 and Huda A and Jordan IK 2009 Epigenetic regulation of mammalian genomes by transposable elements in Ann NY Acad Sci 1178: 276-284). In addition, we have also recently shown that transposable element mediated epigenetic effects on host genomes may not be confined to repressive epigenetic modifications, as emphasized in this review, but also by activating modifications that are recruited to transposable elements in the vicinities of host genes (Huda A et al 2010 Epigenetic histone modifications of human transposable elements: genome defense versus exaptation in Mobile DNA 1:2). There are a couple of other recent papers that are directly related to this topic - and this list is by no means exhaustive - that the authors may wish to have a look at (Rebollo R et al. 2010 Jumping genes and epigenetics: towards new species in Gene 454: 1-7 and Lisch D 2009 Epigenetic regulation of transposable elements in plants Annu Rev Plant Biol. 2009;60:43-66).

**Authors' response: ***This part has been reorganized to integrate this aspect and update citations*.

I agree strongly with the authors' sentiment that transposable elements play critical roles in genome structure, function and evolution. However, some caution is warranted in order to avoid overstating the case. For example, the statement in the abstract that "...since Darwin's theory, transposable elements are maybe the discovery that has changed the most our vision of (genome) evolution." is somewhat overwrought considering that Darwin lacked even the most basic concept of the molecular mechanisms of heredity or any notion whatsoever of what constituted a genome. Indeed, the authors point this very fact out in several places in the manuscript. Thus, they may wish to be more circumspect when placing the impact of transposable elements into the context of evolutionary theory and genome evolution as a whole.

**Authors' response: ***The concerned sentences have been reformulated*

The statement in the introduction that "the core of Darwin's theory was never really questioned." (page 4) is factually inaccurate. The core of this theory has been, and continues to be, continually questioned at a fundamental level. It may be more accurate to state that the core of theory has never been successfully challenged or over-turned.

**Authors' response: ***We agree with this remark and changed the sentence accordingly*.

The authors' imply that biologists were reluctant to accept McClintock's discovery of transposable elements because it did not fit with the 'Central Dogma' (Introduction page 4). But the Central Dogma is a concept from molecular biology that came later, and while the discovery of mobile genetic elements made by McClintock clearly challenged prevailing ideas about how static the genome was, it did not directly address or contradict the Central Dogma. Further on in the same section the Central Dogma is referred to as depicting 'the genome as a linear succession of genes'. Again, the linear 'beads-on-a-string' concept of a static genome is distinct from the Central Dogma.

**Authors' response: ***The confusion between the central dogma and the static genome dogma has been clarified*.

The authors point out an important concept that the evolutionary dynamics of transposable elements occur at two levels: intra-populational, based on the competition between individual organisms, as is the case for static host genes, and intra-genomic based on the competition between individual element copies. This is indeed a critical aspect of transposable element evolution that impacts how the elements affect their host genomes. However, they then go on to posit a third conceptually distinct level based on horizontal transfer. It is well known that elements may be particularly prone to horizontal transfer between species, but it is not clear how and whether this phenomenon entails a third distinct level of transposable element evolutionary dynamics.

**Authors' response: ***This third level become apparent only when an analogy with an ecological concept is considered, which was not clearly stated. The intra-genomic competition may be compared to competition between individuals for the same resource in a unique ecological niche. A TE family in one genome corresponds then to a population. The intra-populational level represents a metapopulation in which TE populations mix by a kind of migration process triggered by sex. By analogy, horizontal escape toward a new genome can be viewed as new ecological niche colonization and represents the extreme case of migration with foundation of a new isolated population and ultimately allopatric speciation. (In comparison a static host gene (allele) will not use the intra-genomic level to expand). We agree that the ability to transfer horizontally does not impact the TE dynamics at the species level, but provides only new seeds for TE expansion in the living world as a whole. The idea has been reformulated, hopefully with more clarity*.

The authors often refer to the conflicting, and seemingly dichotomous, notions of transposable elements as genomic parasites versus the creative or adaptive contributions that the elements make to their host genomes throughout the manuscript. However, these two concepts are not mutually exclusive. In a very nice review on this topic (Kidwell MG and Lisch D 2001 Perspective: transposable elements, parasitic DNA, and genome evolution in Evolution 55:1-24), the authors nicely lay out the idea that transposable elements do not exclusively occupy extreme positions on either end of this dichotomy. They hold, rather, that transposable elements can best be considered as occupying a variety of positions on a dynamic continuum from extreme parasitism to obligate mutualism. This kind of more nuanced perspective would add nicely to the evolutionary role of transposable elements presented here.

**Authors' response: ***We agree with the view of Kidwell and Lisch. However we cannot deny that TE are intrinsically selfish, which was our starting point to further pinpoint facts that actually support other TE-host relationships. The concerned parts have been modified to erase the impression of too clear-cut views*.

Apparently the manuscript was written by a non-native English speaker and it has numerous grammatical errors. The authors should proof the manuscript closely for these and other language related issues or enlist a native English speaking colleague to help with this. For instance, the last sentence in the abstract that reads 'The review attaches to explore ...' does not make sense. The presence of many errors of this kind has the unfortunate effect of obscuring the important message of the manuscript as well as the authors' unique perspective on transposable elements.

**Authors' response: ***The revised text was corrected by a native English speaker*.

## Competing interests

The authors declare that they have no competing interests.

## Authors' contributions

AHV conceived the paper, coordinated the work and drafted the manuscript, ALR designed Figure 3, and critically revised the intellectual content of the whole manuscript, PC helped to draft the last part of the manuscript, TSB was involved in drafting the proposal and critically revised the intellectual content of the last part of the manuscript, JF was involved in drafting the proposal, and helped in critically revising the bacterial data in the manuscript. All authors have read and approved the final manuscript.

## Supplementary Material

Additional file 1 **Supplementary information for Figures 1 and 3**. References for Figure 1, Description of models for Figure 3. Click here for file
