# Supplementary material for: The struggle for life of the genome's selfish architects
Source: Biol Direct. 2011 Mar 17;6:19. doi: 10.1186/1745-6150-6-19 (PMC3072357; doi:10.1186/1745-6150-6-19)
Supplement: Additional file 1 — Supplementary information for Figures 1 and 3. References for Figure 1, Description of models for Figure 3. [file 1745-6150-6-19-S1.PDF]

Supplementary information for Figure 1

References for species for which genome size and percentage of TE are known, cited in figure 1:

Chlamydomonas reinhardtii [1], *Arabidopsis thaliana* [2], *Oryza sativa* [3], *Vitis vinifera* [4], *Populus trichocarpa* [5], *Sorghum bicolor* [6], *Zea mays* [7], *Entamoeba histolytica* [8], *Homo sapiens* [9], *Mus musculus* [10], *Gallus gallus* [11], *Fugu rubripes* [12], *Branchiostoma floridae* [13], *Drosophila melanogaster, Drosophila ananassae* [14], *Anopheles gambiae* [15], *Caenorhabditis elegans* [16], *Saccharomyces cerevisiae* [17], *Nectria haematococca* [18], *Fusarium graminearum* [19], *Tuber melanosporum* [20], *Sulfolobus solfataricus, Sulfolobus acidocaldarius* [21], *Methanothermobacter thermautotrophicus, Halobacterium salinarium, Pyrococcus furiosus* [22], *Bordetella bronchoseptica, Bordetella pertussis* [23], *Gluconacetobacter diazotrophicus* [24], *Prochlorococcus sp.* [25], *Frankia sp. EAN* [26], *Treponema pallidum* [27], *Clostridium difficile* [28], *Streptococcus uberis* [29], *Trypanosoma cruzii* [30], *Trichomonas vaginalis* [31], *Phytophthora infestans* [32], *Theileria parva* [33], *Plasmodium falciparum* [34]

1. Merchant SS, Prochnik SE, Vallon O, Harris EH, Karpowicz SJ, Witman GB, Terry A, Salamov A, Fritz-Laylin LK, Marechal-Drouard L, et al: **The Chlamydomonas genome reveals the evolution of key animal and plant functions.** *Science* 2007, **318:**245-250.
2. The Arabidopsis Genome Initiative: **Analysis of the genome sequence of the flowering plant *Arabidopsis thaliana*.** *Nature* 2000, **408:**796-815.
3. International Rice Genome Sequencing Project: **The map-based sequence of the rice genome.** *Nature* 2005, **436:**793-800.
4. Benjak A, Forneck A, Casacuberta JM: **Genome-wide analysis of the "cut-and-paste" transposons of grapevine.** *PLoS One* 2008, **3:**e3107.
5. Tuskan GA, Difazio S, Jansson S, Bohlmann J, Grigoriev I, Hellsten U, Putnam N, Ralph S, Rombauts S, Salamov A, et al: **The genome of black cottonwood, *Populus trichocarpa* (Torr. & Gray).** *Science* 2006, **313:**1596-1604.
6. Paterson AH, Bowers JE, Bruggmann R, Dubchak I, Grimwood J, Gundlach H, Haberer G, Hellsten U, Mitros T, Poliakov A, et al: **The *Sorghum bicolor* genome and the diversification of grasses.** *Nature* 2009, **457:**551-556.
7. Schnable PS, Ware D, Fulton RS, Stein JC, Wei F, Pasternak S, Liang C, Zhang J, Fulton L, Graves TA, et al: **The B73 maize genome: complexity, diversity, and dynamics.** *Science* 2009, **326:**1112-1115.
8. Lorenzi H, Thiagarajan M, Haas B, Wortman J, Hall N, Caler E: **Genome wide survey, discovery and evolution of repetitive elements in three *Entamoeba* species.** *BMC Genomics* 2008, **9:**595.
9. Lander ES, Linton LM, Birren B, Nusbaum C, Zody MC, Baldwin J, Devon K, Dewar K, Doyle M, FitzHugh W, et al: **Initial sequencing and analysis of the human genome.** *Nature* 2001, **409:**860-921.
10. Waterston RH, Lindblad-Toh K, Birney E, Rogers J, Abril JF, Agarwal P, Agarwala R, Ainscough R, Alexandersson M, An P, et al: **Initial sequencing and comparative analysis of the mouse genome.** *Nature* 2002, **420:**520-562.

11. International Chicken Genome Sequencing Consortium: **Sequence and comparative analysis of the chicken genome provide unique perspectives on vertebrate evolution.** *Nature* 2004, **432:**695-716.
12. Aparicio S, Chapman J, Stupka E, Putnam N, Chia JM, Dehal P, Christoffels A, Rash S, Hoon S, Smit A, et al: **Whole-genome shotgun assembly and analysis of the genome of *Fugu rubripes*.** *Science* 2002, **297:**1301-1310.
13. Putnam NH, Butts T, Ferrier DE, Furlong RF, Hellsten U, Kawashima T, Robinson-Rechavi M, Shoguchi E, Terry A, Yu JK, et al: **The amphioxus genome and the evolution of the chordate karyotype.** *Nature* 2008, **453:**1064-1071.
14. Clark AG, Eisen MB, Smith DR, Bergman CM, Oliver B, Markow TA, Kaufman TC, Kellis M, Gelbart W, Iyer VN, et al: **Evolution of genes and genomes on the Drosophila phylogeny.** *Nature* 2007, **450:**203-218.
15. Holt RA, Subramanian GM, Halpern A, Sutton GG, Charlab R, Nusskern DR, Wincker P, Clark AG, Ribeiro JM, Wides R, et al: **The genome sequence of the malaria mosquito *Anopheles gambiae*.** *Science* 2002, **298:**129-149.
16. Caenorhabditis elegans Sequencing consortium: **Genome sequence of the nematode *C. elegans*: a platform for investigating biology.** *Science* 1998, **282:**2012-2018.
17. Kim JM, Vanguri S, Boeke JD, Gabriel A, Voytas DF: **Transposable elements and genome organization: a comprehensive survey of retrotransposons revealed by the complete *Saccharomyces cerevisiae* genome sequence.** *Genome Res* 1998, **8:**464-478.
18. Coleman JJ, Rounsley SD, Rodriguez-Carres M, Kuo A, Wasmann CC, Grimwood J, Schmutz J, Taga M, White GJ, Zhou S, et al: **The genome of *Nectria haematococca*: contribution of supernumerary chromosomes to gene expansion.** *PLoS Genet* 2009, **5:**e1000618.
19. Ma LJ, van der Does HC, Borkovich KA, Coleman JJ, Daboussi MJ, Di Pietro A, Dufresne M, Freitag M, Grabherr M, Henrissat B, et al: **Comparative genomics reveals mobile pathogenicity chromosomes in Fusarium.** *Nature* 2010, **464:**367-373.
20. Martin F, Kohler A, Murat C, Balestrini R, Coutinho PM, Jaillon O, Montanini B, Morin E, Noel B, Percudani R, et al: **Perigord black truffle genome uncovers evolutionary origins and mechanisms of symbiosis.** *Nature*, **464:**1033-1038.
21. Brugger K, Torarinsson E, Redder P, Chen L, Garrett RA: **Shuffling of Sulfolobus genomes by autonomous and non-autonomous mobile elements.** *Biochem Soc Trans* 2004, **32:**179-183.
22. Filee J, Siguier P, Chandler M: **Insertion sequence diversity in archaea.** *Microbiol Mol Biol Rev* 2007, **71:**121-157.
23. Parkhill J, Sebaihia M, Preston A, Murphy LD, Thomson N, Harris DE, Holden MT, Churcher CM, Bentley SD, Mungall KL, et al: **Comparative analysis of the genome sequences of *Bordetella pertussis*, *Bordetella parapertussis* and *Bordetella bronchiseptica*.** *Nat Genet* 2003, **35:**32-40.
24. Bertalan M, Albano R, de Padua V, Rouws L, Rojas C, Hemerly A, Teixeira K, Schwab S, Araujo J, Oliveira A, et al: **Complete genome sequence of the sugarcane nitrogen-fixing endophyte *Gluconacetobacter diazotrophicus* Pal5.** *BMC Genomics* 2009, **10:**450.
25. Rocap G, Larimer FW, Lamerdin J, Malfatti S, Chain P, Ahlgren NA, Arellano A, Coleman M, Hauser L, Hess WR, et al: **Genome divergence in two**

Prochlorococcus ecotypes reflects oceanic niche differentiation. *Nature* 2003, **424:**1042-1047.
26. Bickhart DM, Gogarten JP, Lapierre P, Tisa LS, Normand P, Benson DR: **Insertion sequence content reflects genome plasticity in strains of the root nodule actinobacterium *Frankia*.** *BMC Genomics* 2009, **10:**468.
27. Seshadri R, Myers GS, Tettelin H, Eisen JA, Heidelberg JF, Dodson RJ, Davidsen TM, DeBoy RT, Fouts DE, Haft DH, et al: **Comparison of the genome of the oral pathogen *Treponema denticola* with other spirochete genomes.** *Proc Natl Acad Sci U S A* 2004, **101:**5646-5651.
28. Sebaihia M, Wren BW, Mullany P, Fairweather NF, Minton N, Stabler R, Thomson NR, Roberts AP, Cerdeno-Tarraga AM, Wang H, et al: **The multidrug-resistant human pathogen *Clostridium difficile* has a highly mobile, mosaic genome.** *Nat Genet* 2006, **38:**779-786.
29. Ward PN, Holden MT, Leigh JA, Lennard N, Bignell A, Barron A, Clark L, Quail MA, Woodward J, Barrell BG, et al: **Evidence for niche adaptation in the genome of the bovine pathogen *Streptococcus uberis*.** *BMC Genomics* 2009, **10:**54.
30. Bhattacharya S, Bakre A, Bhattacharya A: **Mobile genetic elements in protozoan parasites.** *J Genet* 2002, **81:**73-86.
31. Carlton JM, Hirt RP, Silva JC, Delcher AL, Schatz M, Zhao Q, Wortman JR, Bidwell SL, Alsmark UC, Besteiro S, et al: **Draft genome sequence of the sexually transmitted pathogen *Trichomonas vaginalis*.** *Science* 2007, **315:**207-212.
32. Haas BJ, Kamoun S, Zody MC, Jiang RH, Handsaker RE, Cano LM, Grabherr M, Kodira CD, Raffaele S, Torto-Alalibo T, et al: **Genome sequence and analysis of the Irish potato famine pathogen *Phytophthora infestans*.** *Nature* 2009, **461:**393-398.
33. Gardner MJ, Bishop R, Shah T, de Villiers EP, Carlton JM, Hall N, Ren Q, Paulsen IT, Pain A, Berriman M, et al: **Genome sequence of *Theileria parva*, a bovine pathogen that transforms lymphocytes.** *Science* 2005, **309:**134-137.
34. Gardner MJ, Hall N, Fung E, White O, Berriman M, Hyman RW, Carlton JM, Pain A, Nelson KE, Bowman S, et al: **Genome sequence of the human malaria parasite *Plasmodium falciparum*.** *Nature* 2002, **419:**498-511.

Supplementary information for Figure 3

The dynamics for the transposition model are described by

$$a(t+1) = a(t) + a(t).[s/(1+s(a(t)+n(t))) + u.(\exp(-n(t)) + a(t)/(a(t)+n(t)) - v] - m.a(t)$$

$$n(t+1) = n(t) + n(t).[s/(1+s(a(t)+n(t))) + u.(1-\exp(-a(t))).a(t)/(a(t)+n(t)) - v] + m.a(t)$$

where a(t) is the number of active copies at generation t, n(t) is the number of inactive copies at generation t, s is the deleterious effect of a TE insertion, u the transposition rate, v the deletion rate, and m the mutation rate from an active to an inactive copy. The parameters used in the figure are: u = 0.015, v=0.001, s=-0.005, m=0.002, a(0) = 1, and n(0) = 0.

The prey-predator model is a discrete version of the Lotka-Volterra setting with density dependence:

$$x(t+1) = x(t) + A\, x(t) - B\, x(t)\, y(t)$$

$$y(t+1) = y(t) + D\, x(t)\, y(t) - G\, y(t) + E\, y(t)\, y(t)$$

x(t) and y(t) correspond to the number of preys and predators at generation t, A is the growth rate of preys in absence of predators, B is the predation rate, D is the growth rate of predators per prey catch, G is the death rate of predators, and E is a density-dependence parameter of predators. Parameters used in the figure are A=0.005, B=0.002, D=0.002, G=0.002, and E=-0.003.
